# Integrated Single‐Cell and Spatial Analysis Reveals a Metabolic‐Immune Axis Driving Aortic Dissection

**DOI:** 10.1002/advs.75509

**Published:** 2026-05-10

**Authors:** Jing Tao, Huanjie Yang, Jiahui Yong, Xueting Chen, Qiang Zhao, Xueli Wu, Lei Yan, Li Pang, Fan Luo, Mengjun Yu, Shanshan Pan, Deyang Li, Rouxi Chen, Juan Wang, Zhensheng Dong, Fan Yang, Yue Wang, Yang Chen, Hongjian Zheng, Zhimin Yang, Zijie Wang, Karsten Kristiansen, Hui Peng, Xiaodong Fang, Juan Shen, Yining Yang

**Affiliations:** ^1^ Department of Cardiology People's Hospital of Xinjiang Uygur Autonomous Region Urumqi Xinjiang China; ^2^ Xinjiang Key Laboratory of Cardiovascular Homeostasis and Regeneration Research Urumqi Xinjiang China; ^3^ BGI Research Shenzhen Guangdong China; ^4^ College of Life Sciences University of Chinese Academy of Sciences Beijing China; ^5^ Zhuhai UM Science & Technology Research Institute Zhuhai Guangdong China; ^6^ IDigital Biotechnology Sanya Hainan China; ^7^ BGI Research Qingdao Shandong China; ^8^ BGI Research Sanya Hainan China; ^9^ BGI Research Beijing China; ^10^ State Key Laboratory of Dampness Syndrome of Chinese Medicine The Second Affiliated Hospital of Guangzhou University of Chinese Medicine Guangzhou Guangdong China; ^11^ Laboratory of Integrative Biomedicine Department of Biology University of Copenhagen Copenhagen Denmark

**Keywords:** aortic dissection, enolase 1, fibroblast, macrophage, *MIF*, single‐cell RNA sequencing, spatial transcriptomics

## Abstract

Although single‐cell studies have profiled diseased aorta, mechanisms driving aortic dissection (AD) remain largely elusive owing to limited cohorts. Here, we integrate single‐cell and spatial transcriptomic data from 110 thoracic aortic samples (80 individuals; control, aneurysm, dissection; 767 018 high‐quality cells) to generate a comprehensive thoracic‐aorta cellular—molecular atlas. We identify an elastin‐rich fibroblast subset (Fibro_C1_FBN1+; *FBN1*, *MFAP5*, *LOX*) that declines with age and is markedly depleted in AD, linking fibroblast loss to increased aortic wall vulnerability and dissection risk. Vascular smooth muscle cells (vSMCs) undergo *ENO1*‐driven glycolytic reprogramming under hypoxia, lose contractility and adopt a synthetic, *MIF*‐secreting phenotype that engages macrophage receptors to promote macrophage recruitment and pro‐inflammatory polarization, leading aggregated macrophages to upregulate proteolytic and fibrinolytic pathways and thereby accelerate extracellular‐matrix degradation. In vitro and in vivo, *ENO1* knockdown inhibits vSMC switching, reduces macrophage inflammation, and slows AD progression. This stromal‐immune axis suggests potential therapeutic targets in AD.

## Graphical abstract

Single‐cell and spatial profiling of 110 human thoracic aortic samples reveals a stromal–immune circuit driving aortic dissection. An elastin‐rich fibroblast subset is depleted with age and markedly reduced in disease, weakening aortic wall integrity. vSMCs undergo *ENO1*‐driven metabolic reprogramming into a MIF‐secreting state that activates macrophages and accelerates extracellular matrix degradation, suggesting therapeutic vulnerability.

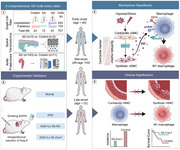

## 1 Introduction

The thoracic aorta's trilaminar wall (intima, media, adventitia), reinforced by elastic fibers and vSMCs, maintains structural integrity and functional resilience under constant pulsatile stress, and disruption of this architecture can precipitate catastrophic vascular failure. Among thoracic aortic pathologies, aortic dissection represents one of the most devastating cardiovascular emergencies, characterized by a sudden intimal tear that allows blood to enter and split the medial layer, forming a false lumen and compromising flow to major aortic branches, potentially leading to acute aortic regurgitation, myocardial ischemia, or fatal rupture [1, 2, 3, 4, 5, 6, 7].

Recent advances in single‐cell RNA sequencing (scRNA‐seq) and spatial transcriptomics have provided unprecedented insights into the cellular diversity of the human thoracic aorta and revealed key mechanisms underlying thoracic aortic disease, including vSMC phenotypic switching, endothelial dysfunction, macrophage‐driven inflammation, ECM degradation, and lymphocyte‐mediated immune amplification [8, 9, 10, 11, 12, 13, 14, 15, 16, 17, 18, 19]. However, most existing studies are single‐center with small cohorts, resulting in batch effects, limited statistical power, and inconsistent cell‐type annotations. Many datasets also lack comprehensive cell coverage, focusing on selected immune or stromal subsets, which hinders identification of conserved disease mechanisms [20, 21, 22, 23]. To address these limitations, constructing a comprehensive, integrative single‐cell atlas similar to existing efforts in the lung, heart, and vascular endothelium is essential to unify disparate datasets, enable robust cross‐study comparisons, and define conserved and disease‐specific molecular programs [24, 25, 26].

Here, we constructed a large‐scale, multi‐center single‐cell and spatial transcriptomic atlas of the human thoracic aorta, comprising 767 018 high‐quality cells from 110 samples across 80 individuals, including controls, aortic aneurysm (AA) and AD. By integrating public and newly generated datasets across multiple sequencing platforms, we minimized center‐specific biases, enhance detection of rare and transitional cell states, and improved cross‐cohort robustness. This resource established the most comprehensive reference to date for human thoracic aortic biology and provides a foundation to dissect molecular and cellular mechanisms underlying aneurysm and dissection progression.

## 2 Results

### 2.1 A Comprehensive Single‐Cell Atlas of Human Thoracic Aortic Diseases

**FIGURE 1:**
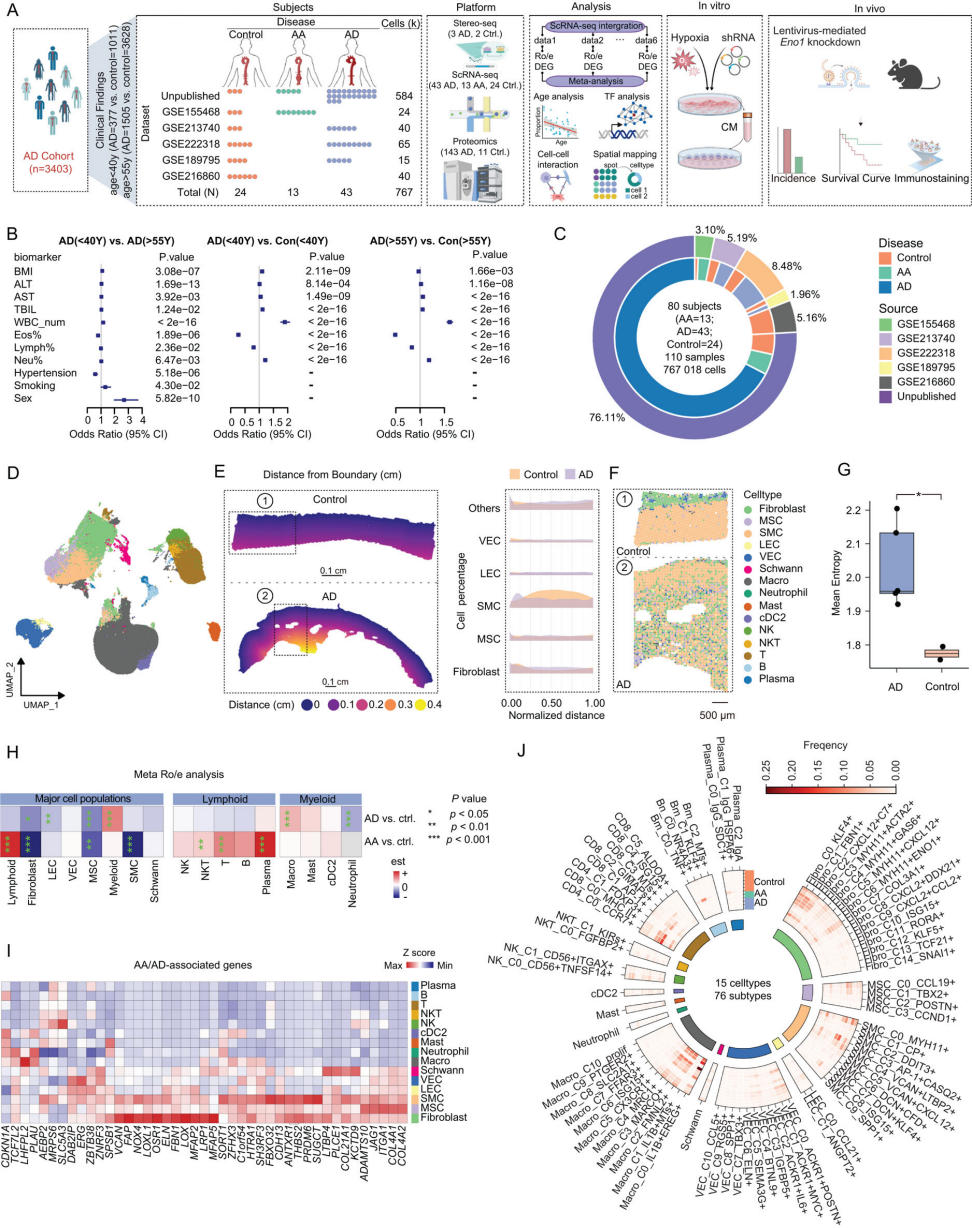
Overview of a multicenter human aortic vascular cell atlas in AD, AA and control samples. (A) Schematic of the experimental workflow: tissue collection from patients with AD, AA, and controls; followed by scRNA‐seq,Stereo‐seq, proteomics, immunostaining, ex vivo cell culture, and validation in murine models. Illustrations created using BioRender. (B) Clinical phenotype distributions across age groups. *P*‐values and odds ratios (OR) derived from logistic regression, adjusting for covariates (AD vs. control: age and sex; younger vs. older AD: sex only). OR denotes change in odds for binary outcomes or average change in log‐odds per unit increase in continuous predictors. (C) Proportions of cell types across disease states in each dataset (including novel data generated in this study). (D) UMAP visualization of all high‐quality single‐cell transcriptomic data (*n* = 110 samples) showing clustering by cell type. (E) Left: Spatial distribution of regions at increasing distances form the arterial adventitia.Right: Spatial distribution of cell2location‐derived cell‐type weights across distinct distance‐defined regions of the arterial media; lower distance values indicate closer proximity to the adventitia. (F) Spatial distribution of cell types in the AD and Control groups, with each spot assigned to the cell type of highest probability as determined by Cell2location. (G) Shannon entropy of cell‐type distributions across AA, AD, and control states in spatial transcriptomic data. (H) Meta‐analysis of cell‐type enrichment: R o/e (ratio of observed to expected proportions) shown per cell type. Pooled effect sizes (log ratios) obtained via random‐effects meta‐analysis (REML, metafor); two‐sided *p* values from Wald‐type tests. Significance levels are indicated as ^*^ *p* < 0.05, ^**^ *p* < 0.01, ^***^ *p* < 0.001, ^****^ *p* < 0.0001. (I) Heatmap of Z‐score levels of previously reported AD/AA‐associated genes across cell types. (J) Circular diagrams depicting subtype frequency distributions under different disease conditions. AD, aortic dissection; AA, aortic aneurysm; OR, odds ratio; scRNA‐seq, single‐cell RNA sequencing; UMAP, uniform manifold approximation and projection; Ro/e, ratio of observed to expected; REML, restricted maximum‐likelihood; WBC, white blood cell; Eos%, eosinophil percentage; Lymph%, lymphocyte percentage; Neu%, neutrophil percentage; cDC2, conventional dendritic cell type 2; SMC, smooth muscle cell; VEC, vascular endothelial cell; LEC, lymphatic endothelial cell; MSC, mesenchymal cell. [Correction added on 12 May 2026 after first online publication: Updated figure 1 is available in this version.]

During the period 2004–2024, 3403 subjects were diagnosed with AD at our center, comprising 377 aged younger than 40 years (early‐onset AD) and 1505 aged older than 55 years (late‐onset AD) (Figure 1A). To systematically characterize the clinical features of AD, we compared AD patients with matched controls by age, gender, and region (Figure 1B and Table S1). The results showed that both early‐onset and late‐onset AD patients exhibited elevated inflammatory markers, including D‐dimer, C‐reactive protein (CRP), white blood cell (WBC) count, liver enzymes (alanine transaminase [ALT], aspartate transaminase [AST], and total bilirubin), and neutrophil proportions, alongside decreased eosinophil and lymphocyte levels. These findings indicated ongoing inflammation and immune suppression in these patients [27, 28, 29, 30]. Notably, despite these shared features, distinct differences emerged between the two groups. The early‐onset AD group had a significantly higher WBC count than the late‐onset group (*p* < 2e‐16) and a markedly elevated male‐to‐female ratio (5.98 vs. 2.26, *p* = 5.80e‐11, 95% CI = 2.65 [1.96–3.64]).

To systematically and comprehensively characterize the cellular heterogeneity of the human thoracic aorta and elucidate the pathogenic mechanisms of AA and AD, we constructed an integrated single‐cell and spatial transcriptomic atlas (Figure 1A; Figure S1). This integrative analysis was primarily driven by the newly generated single‐cell and spatial transcriptomic datasets from this study, complemented by five previously published single‐cell transcriptomic datasets (GSE155468, GSE189795, GSE213740, GSE216860 and GSE222318) (Figure 1A) [16, 31, 32, 33]. The study included a total of 80 subjects and 110 tissue samples, comprising 24 controls, 13 AA subjects, and 43 AD subjects (Figure 1C; Figure S2A and Table S2). After rigorous quality control and batch effect correction, we obtained 767 018 high‐quality cells, 76.11% of which were newly generated in this study (Figure 1C; Figure S2B and Table S3). No significant batch effect was observed across samples from different datasets, ages, sexes, or disease states (Figure S2C). Unbiased clustering identified 15 major cell populations (Figure 1D; Figure S2D and Table S4). Spatial transcriptomic analysis and histopathological analysis confirmed the loss of cellular organization in AD, resulting in severe disarray at the rupture site, as evidenced by a significant increase in spatial entropy, which correlated with histopathological damage (Figure 1E–G; Figure S2E).

**FIGURE 2:**
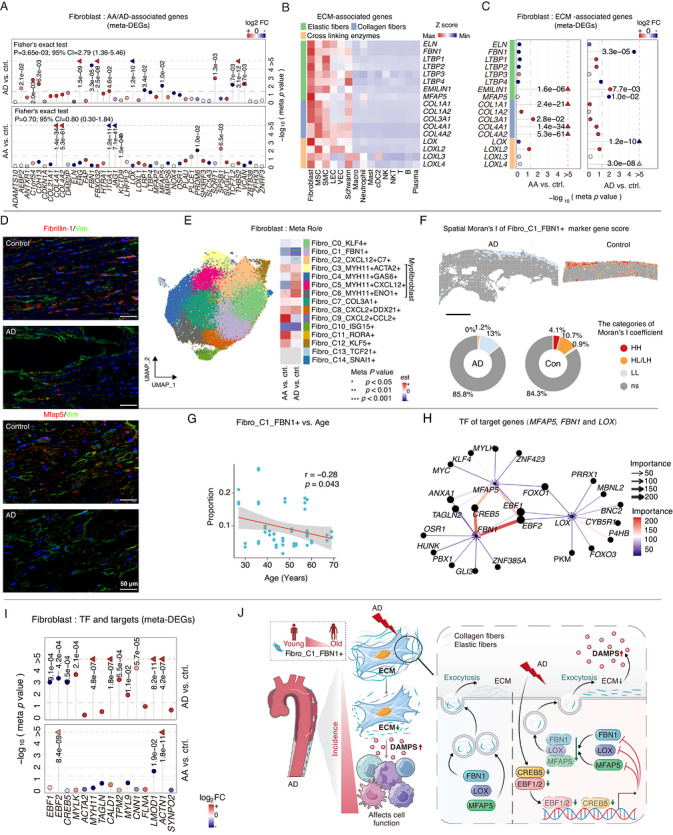
Heterogeneity of aortic fibroblasts across disease states. (A) Meta‐analysis of differential expression of AD‐ and AA‐associated genes in fibroblasts. Log2 fold changes (log2FC) and meta‐analysis *p* values were computed using a random‐effects model across datasets. (B) Heatmap of ECM‐related gene Z‐score across cell types. (C) Meta‐analysis of differential expression of ECM‐related genes in fibroblasts across disease states. (D) Immunofluorescence images of human aortic tissues showing *FBN1* (green) and *MFAP5* (red) expression across disease states. (E) UMAP embedding of fibroblast subtypes and corresponding meta Ro/e. Significance levels are indicated as ^*^ *p* < 0.05, ^**^ *p* < 0.01, ^***^ *p* < 0.001, ^****^ *p* < 0.0001. (F) Local Moran's I hotspot clusters of the Fibro_C1_FBN1+ subtype marker gene score (*FBN1*, *MFAP5*, and *ELN*) in representative spatial transcriptomic samples, and the circular plot showing the proportion of local Moran's I hotspot clusters across all samples. Scale bar = 2 mm. Pie charts displaying the proportion of local Moran's I hotspot clusters for the Fibro_C1_FBN1+ subtype (markers: *FBN1*, *MFAP5*, *ELN*) in spatial transcriptomics data from selected samples. (G) Correlation between proportions of fibroblast subtypes and age across samples in unpublished data, assessed by Pearson's r; red line indicates linear regression with 95% confidence interval. (H) Transcriptional regulatory network targeting *MFAP5*, *FBN1*, and *LOX*. Edge width and color indicate regulatory importance; node size reflects degree centrality; arrows denote regulatory direction. (I) Meta‐analysis of differential expression of transcription factors targeting *MFAP5*, *FBN1*, and *LOX* across disease states. (J) Hypothetical model depicting how fibroblasts may potentially influence AD progression; illustration created with BioRender.

To ensure robust cross‐dataset comparison, we assessed cellular enrichment using the observed‐to‐expected cell‐type ratio (Ro/e) followed by meta‐analysis. Meta‐analysis demonstrated a pronounced enrichment of myeloid cells in AD (46.90% vs. 16.90% in controls), with macrophages being particularly overrepresented, whereas AA specimens showed a significant increase in lymphoid cells (44.92% vs. 15.16%) (Figure 1H; Figure S3A–C), consistent with prior studies [9, 14, 31]. Beyond these disease‐specific alterations in immune cell abundance, analysis of the core cell populations revealed distinct differences in cell type‐specific drug targets (Figure S3D). Furthermore, known AA/AD susceptibility genes were predominantly enriched in non‐immune stromal populations (Figure 1I). To achieve a higher‐resolution view of pathogenesis, we performed subclustering analysis on each major cell type, which identified 76 distinct subtypes for subsequent analyses (Figure 1J; Figure S4 and Table S5).

### 2.2 Fibroblast‐Driven Elastic Fiber Failure Over Collagen in AD

Fibroblasts residing in the adventitia of the thoracic aorta represent the key cellular population responsible for ECM production and vascular repair following injury [34, 35]. Among the previously reported AA/AD susceptibility genes, we observed a strong enrichment of differentially expressed genes in vSMCs in AD compared with controls (*p* = 3.65e‐03; 95% CI = 2.79 [1.36‐5.46]), further supporting an important role of fibroblasts in AD pathogenesis (Figure 2A). Compared with controls, fibroblasts from AD subjects did not show significant changes in the expression of collagen‐related genes (*COL1A1*, *COL1A2*, *COL3A1*, *COL4A1*, *COL4A2*), whereas the expression of elastin fiber‐associated genes, including *FBN1*, *MFAP5*, and the crosslinking enzyme *LOX*, was significantly reduced in AD fibroblasts (Figure 2B–D) [36, 37].

To delineate the mechanisms underlying the selective downregulation of elastic fiber genes despite preserved collagen expression, we performed fine‐grained fibroblast subclustering and identified 15 transcriptionally distinct subtypes (Figure 2E). Among these subtypes, the Fibro_C1_FBN1+ subtype, which is defined by elevated expression of elastic fiber‐related genes, was markedly depleted in AD patients, exhibited lower senescence‐index scores, and was associated with higher survival in individuals with high *FBN1* expression (Method), and this subtype also showed a pronounced age‐dependent decline (Figure 2E–G; Figure S5A‐G; Table S5). The myofibroblast subtype, defined by upregulation of smooth muscle markers (*ACTA2*, *MYH11*), was significantly expanded in AD, showed higher hUSI scores, lacked age dependency, and displayed a pathogenic association consistent with previous cardiovascular studies (Figure 2E; Figure S5A,C,E–G and Table S5) [38, 39].

To identify regulatory factors driving the reduction of Fibro_C1_FBN1+ and expansion of myofibroblasts, we applied SCENIC‐based transcriptional network inference. In AD fibroblasts, transcription factors *EBF1*/*2* and *CREB5* were markedly downregulated relative to controls and AA, likely leading to decreased expression of their downstream targets *FBN1*, *MFAP5*, and *LOX* (Figure 2H–J). This dysregulation may impair elastic fiber synthesis while leaving collagen production relatively unaffected, thereby compromising ECM elasticity and repair capacity, weakening the aortic wall, and predisposing to dissection. Concurrently, *MYLK* was significantly upregulated in AD fibroblasts and acted as a transcriptional activator of contractility‐associated genes (*ACTA2*, *MYH9*, *MYH11*, *TAGLN*), potentially promoting pathogenic fibroblast‐to‐myofibroblast transition and exacerbating disease progression (Figure 2I; Figure S5H‐,I).

To explore potential therapeutic avenues, we employed the drug2cell computational framework, which predicts drug‐protein interactions based on known target networks, to identify candidate compounds modulating Fibro_C1_FBN1+ or myofibroblast subtypes. Notably, aminocaproic acid, an antifibrinolytic agent, emerged as a potential modulator [40, 41, 42, 43]. This compound competitively binds to the lysine‐binding sites of plasminogen, inhibiting its conversion to plasmin and thereby reducing ECM degradation. Although primarily used to manage hyperfibrinolysis‐induced bleeding, its capacity to suppress ECM proteolysis suggests a previously unrecognized therapeutic potential in AD, particularly in mitigating vascular ECM destruction and intramural hemorrhage (Figure S5J).

### 2.3 Metabolic Reprogramming Drives vSMCs Dedifferentiation and Contributes to AD

**FIGURE 3:**
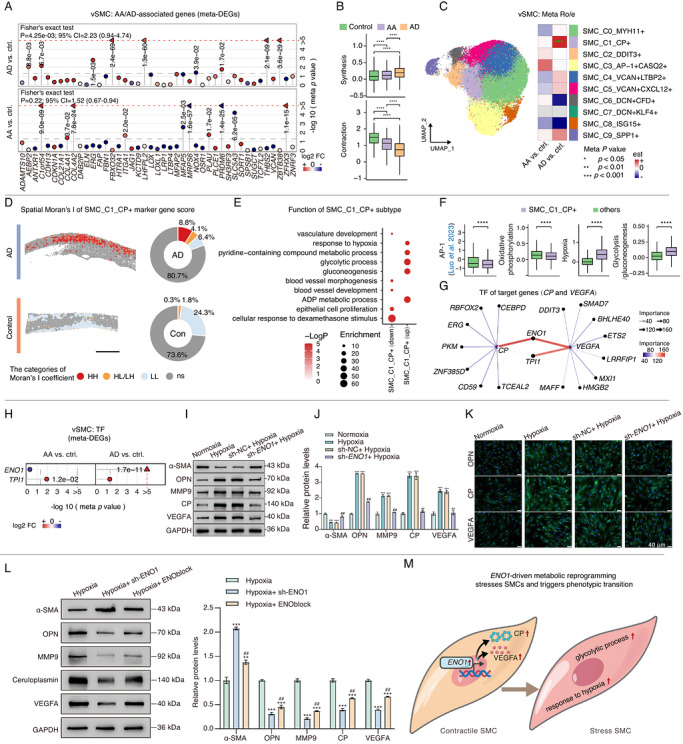
Heterogeneity of vSMCs across disease states. (A) Meta‐analysis of differential expression of AD‐ and AA‐associated genes in vSMCs. B) Box plots of vSMCs gene scores for synthesis vs. contraction programs across disease states. Pairwise comparisons were performed using Wilcoxon rank‐sum tests; significance levels are indicated as ^*^ *p* < 0.05, ^**^ *p* < 0.01, ^***^ *p* < 0.001, ^****^ *p* < 0.0001. (C) UMAP visualization of vSMC subtypes alongside meta Ro/e (observed‐to‐expected cell‐type proportion ratio). Pooled effect sizes (log ratios) derived via random‐effects meta‐analysis (REML, metafor); two‐sided *p* values from Wald‐type tests. Significance levels are indicated as ^*^ *p* < 0.05, ^**^ *p* < 0.01, ^***^ *p* < 0.001, ^****^ *p* < 0.0001. (D) Local Moran's I hotspot clusters of the SMC_C1_CP+ subtype marker gene score (*CP*, *NDRG1*, *VEGFA*) in representative spatial transcriptomic samples, and the circular plot showing the proportion of local Moran's I hotspot clusters across all samples. Scale bar = 2 mm. (E) Gene Ontology (GO) enrichment pathways for upregulated and downregulated marker genes of SMC_C1_CP+. (F) Boxplots of hypoxia, AP‐1, oxidative phosphorylation and glycolysis/gluconeogenesis scores in SMC_C1_CP+ vs. non‐SMC_C1_CP+ vSMCs. Pairwise comparisons via Wilcoxon rank‐sum test. Significance levels are indicated as ^*^ *p* < 0.05, ^**^ *p* < 0.01, ^***^ *p* < 0.001, ^****^ *p* < 0.0001. (G) Transcriptional regulatory network targeting *CP* and *VEGFA*. Edge width and color denote regulatory importance; node size indicates degree centrality; arrows show regulation direction. (H) Meta‐DEG analysis of transcription factors targeting *CP* and *VEGFA* across disease states. (I,J) Western blot analysis of key proteins under Normoxia, Hypoxia, sh‐NC+Hypoxia, and sh‐*ENO1*+Hypoxia conditions. Examined markers include contractile vSMC marker α‐SMA, synthetic vSMC markers OPN and MMP9, and SMC_C1_CP+ markers Ceruloplasmin and VEGFA. Statistical comparisons via one‐way ANOVA with Tukey's post‐hoc test: ^***^ *p* < 0.001 vs. Normoxia; ^**^ *p* < 0.01 vs. Normoxia; ##*p* < 0.01 vs. sh‐NC+Hypoxia. (K) Immunofluorescence imaging of synthetic vSMC marker OPN and SMC_C1_CP+ markers Ceruloplasmin and VEGFA under varying conditions. (L) Western blot analysis of key phenotypic markers under Hypoxia, Hypoxia + sh‐*ENO1*, and Hypoxia + ENOblock conditions, including synthetic markers (OPN, MMP9) and SMC_C1_CP+ markers (CP, VEGFA). (M) Schematic illustrating the mechanism of vSMC phenotypic switching from contractile to synthetic states; illustrations created using BioRender. For panels A and H, meta‐DEG analysis using random‐effects model; log2FC indicates integrated expression change and direction; meta‐analysis *p* values reflect pooled significance.

VSMCs, the predominant effector cells maintaining aortic structure and function, are highly plastic and can switch to a dedifferentiated synthetic phenotype in response to hypoxia, injury or other stress, thereby promoting arterial degeneration and dissection [9, 44, 45]. Among the previously reported AA/AD susceptibility genes, we observed a strong enrichment of DEGs in vSMCs when comparing AD to control samples (*p* = 4.25e‐03; 95% CI = 2.23 [0.94–4.74]), further supporting the important role of vSMCs in AD pathogenesis (Figure 3A). Compared to controls, vSMCs from AD patients exhibited a significant reduction in contractile markers and a marked elevation in synthetic markers, notably osteopontin (*SPP1*), indicating a phenotypic transition toward a synthetic state (Figure 3B; Figure S6A).

To further dissect vSMC heterogeneity, we performed high‐resolution subtype analysis and identified ten transcriptionally distinct subtypes (Figure 3C). Among the identified subpopulations, the age‐independent subtype SMC_C1_CP+, which was markedly expanded in the AD group, exhibited elevated senescence index scores, and exploratory analyses of GEPIA‐based TCGA pan‐cancer cohorts further showed that elevated *CP* expression was associated with poorer disease‐free survival, indirectly supporting the potential biological relevance of *CP* in disease‐associated prognostic programs despite the non‐AD‐specific nature of this analysis (Figure 3C,D; Figure S6B–G). This subtype exhibited strong enrichment of hypoxia‐ and glycolysis/gluconeogenesis‐associated genes, reflecting a hypoxia‐induced metabolic reprogramming toward enhanced glycolytic activity, in line with previous findings (Figure 3E,F) [46, 47]. In contrast, it showed significant downregulation of *AP‐1* and oxidative phosphorylation‐related genes, indicating functional divergence from previously described disease‐associated vSMC states and suggesting a potentially novel pathogenic mechanism (Figure 3F) [9].

To explore the factor driving vSMCs toward the SMC_C1_CP+ subtype, we performed transcription‐factor analysis and found that vSMCs in the AD group, compared with controls and the AA group, specifically up‐regulated the glycolytic enzyme gene *ENO1*, which regulates the expression of *CP* and *VEGFA*, thereby exhibiting distinct disease‐specific heterogeneity (Figure 3G,H). These results indicate that metabolic reprogramming of vSMCs, characterized by enhanced glycolytic flux, promotes their transition from a contractile to a synthetic SMC_C1_CP+ phenotype, with *ENO1* likely contributing to the metabolic remodeling that facilitates this phenotypic shift (Figure 3G,H). In the pathological context of chronic inflammation and vascular hypoxia, which are major mechanisms underlying AD, we further confirmed experimentally that hypoxic stress markedly increases *ENO1* expression at both the transcript and protein levels, supporting its critical role in hypoxia‐induced phenotypic remodeling of vSMCs (Figure S6H–J) [14, 48, 49, 50].

To further elucidate the mechanistic role of *ENO1* in metabolism‐driven phenotypic reprogramming of vSMCs, we designed four experimental conditions: normoxia, hypoxia, sh‐NC+hypoxia, and sh‐*ENO1*+hypoxia (Figure 3I–K; Figure S6K). Under hypoxic conditions, the contractile marker α‐SMA was markedly down‐regulated, while the synthetic markers *OPN* and *MMP9* together with the disease‐associated subtype markers *Ceruloplasmin* and *VEGFA* were significantly up‐regulated and vSMC migratory capacity increased, indicating that hypoxia drives a pronounced transition of vSMCs toward the synthetic SMC_C1_CP+ phenotype, whereas knock‐down of *ENO1* substantially delayed this phenotypic conversion. Mechanistically, *ENO1* was found to modulate vSMC phenotypes through its dual roles as a metabolic enzyme and a transcriptional regulator (Figure 3L). These findings identified *ENO1* as a pivotal mediator that coordinated the hypoxia‐induced transition of vSMCs from a contractile to a synthetic state. This mechanism appeared distinct from the previously reported tumour necrosis factor‐OXPHOS‐*AP‐1* axis, suggesting that *ENO1*‐driven metabolic reprogramming represents an alternative and previously unrecognized pathogenic pathway contributing to the development of AD (Figure 3M). However, Drug2Cell analysis revealed that no existing pharmacological agents target the metabolic pathway associated with this disease‐relevant vSMC subtype. Current drugs primarily act on the *VEGFA* signaling axis rather than on *ENO1* or related glycolytic nodes, underscoring a potential therapeutic gap in targeting metabolic reprogramming during AD progression (Figure S6L) [47, 51, 52, 53, 54, 55, 56, 57, 58, 59, 60, 61, 62].

### 2.4 Macrophage‐Driven Inflammatory Remodeling Shapes the Vascular Microenvironment in AD

**FIGURE 4:**
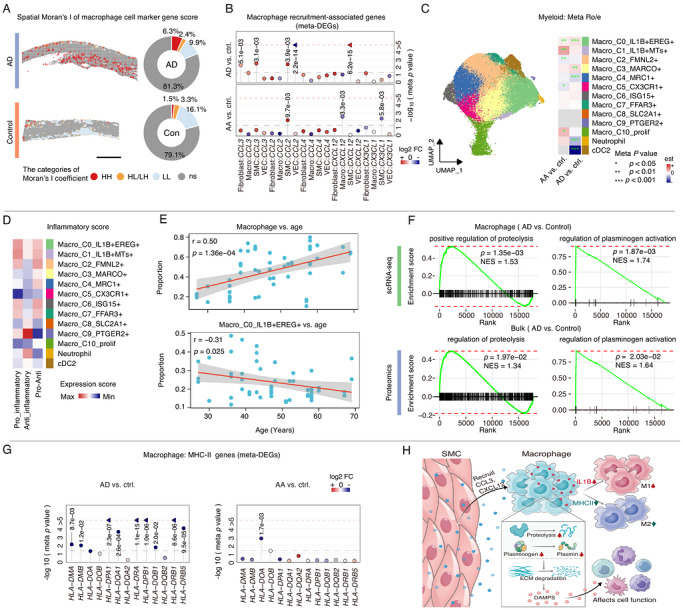
Macrophages adversely impact AD progression through multiple mechanisms. (A) Local Moran's I hotspot clusters of the macrophage marker gene score (*C1QA*, *C1QB*, *CD163*, *CD14*) in representative spatial transcriptomic samples, and the circular plot showing the proportion of local Moran's I hotspot clusters across all samples. Scale bars = 2 mm. (B) Meta‐analysis of AD‐ and AA‐related gene expression differences in macrophage. (C) UMAP embedding of myeloid cell subtypes and corresponding meta Ro/e. Significance levels are indicated as ^*^ *p* < 0.05, ^**^ *p* < 0.01, ^***^ *p* < 0.001, ^****^ *p* < 0.0001. (D) Heatmap of pro‐inflammatory vs. anti‐inflammatory gene scores across myeloid cell types. (E) Correlation plots showing how proportions of myeloid cells (overall and by subtype) vary with sample characteristics in unpublished data. (F) GSEA results based on macrophage single‐cell RNA‐seq and aortic proteomic datasets; normalized enrichment scores (NES) are presented. (G) Meta‐analysis of differential expression of MHC II genes in macrophages, as in D. (H) Hypothetical model depicting how macrophages may potentially influence AD progression; illustration created with BioRender.

Numerous studies show that medial and adventitial macrophage infiltrates in dissected aorta secrete pro‐inflammatory cytokines and matrix‐degrading proteases, accelerating ECM and elastic fibre disruption [14, 16, 17, 63, 64, 65, 66, 67, 68, 69]. Although reported AA/AD risk genes were not significantly enriched among differentially expressed genes in AD (*p* = 0.15; 95% CI = 1.68 [0.76–3.41]), our single‐cell RNA data revealed a marked increase in macrophage abundance in AD (43.89%) compared to controls (15.21%), a finding further validated by spatial transcriptomics (Figure 4A,B; Figure S7A–C). To explore drivers of macrophage accumulation in AD, we performed a meta‐analysis of recruitment‐related chemokines and found that vSMCs from AD upregulate *CCL2*/*CCL3*/*CXCL12*, which likely enhance macrophage chemotaxis via their cognate receptors, amplify local inflammation, and fuel disease progression (Figure 4B). Notably, endothelial cells, which are often considered a major source of monocyte‐recruiting signals, did not exhibit similar chemokine upregulation in our dataset, diverging from previous reports [16, 64, 70, 71, 72].

To delineate macrophage heterogeneity, we performed subclustering of myeloid cells and identified 11 macrophage subtypes, one neutrophil cluster, and one conventional dendritic cell (cDC) population. The pro‐inflammatory Macro_C0_IL1B+EREG+ subtype was markedly expanded in AD compared with controls (Figure 4C,D; Figure S7C,D). Intriguingly, although the overall macrophage population increased with age in the AD group, the relative abundance of Macro_C0_IL1B+EREG+ declined, indicating that its inflammatory activity may be preferentially engaged in younger patients, potentially contributing to early‐onset vascular injury (Figure 4E).

Functional enrichment analyses revealed that macrophages in AD specifically upregulated pathways related to “positive regulation of proteolysis” and “regulation of plasminogen activation” reflecting an enhanced ECM‐degrading activity (Figure 4F; Figure S7E). Proteomic data from aortic tissue further corroborated this trend (Figure 4F). Conversely, AD macrophages exhibited downregulation of antigen‐presentation pathways, including “antigen processing and presentation of peptide antigen via MHC class II” accompanied by reduced expression of MHC II genes, suggesting compromised immune surveillance and clearance of vascular injury, findings that contrast with several prior studies (Figure 4G; Figure S7E) [15, 17]. Collectively, these findings delineate a pathological cascade wherein macrophage‐driven ECM degradation and impaired antigen presentation synergistically promote sustained inflammation and structural disintegration of the aortic wall (Figure 4H). These changes may create a self‐reinforcing loop of matrix breakdown and damage‐associated molecular pattern (DAMP) release, fueling persistent inflammation and medial layer rupture (Figure 4H).

Finally, to identify potential therapeutic interventions, we applied Drug2cell to the pro‐inflammatory macrophage subtype expanded in AD. The analysis predicted that the PPARγ agonist rosiglitazone may suppress M1 polarization and attenuate macrophage inflammatory programs, while the ACE inhibitor lisinopril, beyond its antihypertensive effects, could indirectly mitigate myeloid inflammatory activation (Figure S7F) [73, 74, 75, 76]. Together, these results suggest that pharmacological modulation of macrophage‐driven inflammation may represent a promising therapeutic avenue for preventing or slowing AD progression.

### 2.5 Metabolic Reprogramming via *ENO1* Drives *MIF*‐Mediated Macrophage Retention and Inflammatory Activation in AD

**FIGURE 5:**
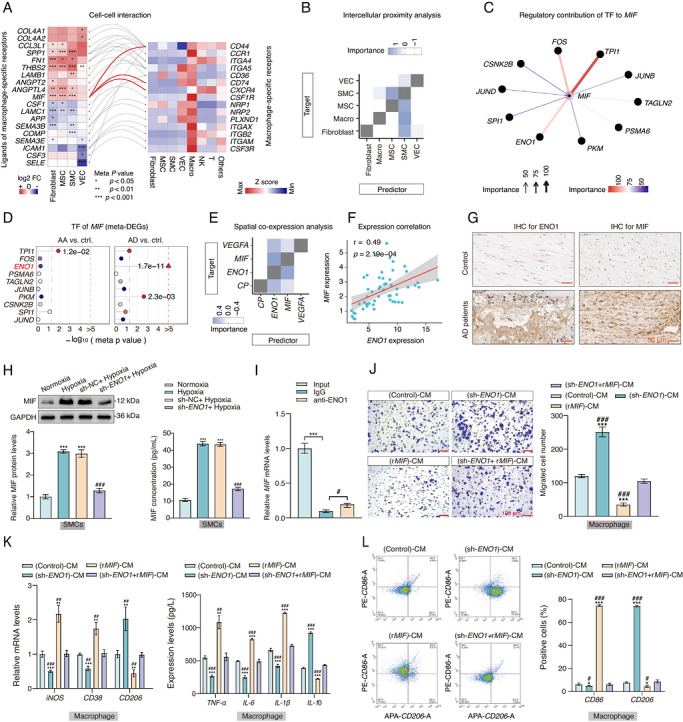
Non‐immune cells influence macrophage inflammatory and migration phenotype via the *MIF*‐*ENO1* axis. (A) Heatmap combining meta‐analysis‐derived ligands that are differentially expressed in non‐immune cells of disease samples with the Z‐score profiles of their corresponding macrophage‐specific receptor genes. Significance levels for ligand differential expression are denoted as ^*^ *p* < 0.05, ^**^ *p* < 0.01, ^***^ *p* < 0.001, and ^**^ *p* < 0.0001. (B) Spatial localization analysis with MISTy applied to cell2location deconvolution outputs from spatial transcriptomics. (C) Regulatory network of transcription factors targeting *MIF*; edge width and color indicate regulatory importance, node size reflects degree centrality, and arrows denote regulatory direction. (D) Meta‐analysis of differential expression of transcription factors targeting *MIF* in vSMCs. (E) Spatial colocalization of *MIF* and *ENO1* with SMC_C1_CP+ markers (*CP*, *VEGFA*) in human aortic tissue. (F) Spearman correlation of *ENO1* and *MIF* expression at the sample level in vSMCs. (G) Immunohistochemistry (IHC) for ENO1 and MIF expression in human aortic tissue. (H) Protein levels and concentrations of MIF in SMC supernatants under normoxia, hypoxia, sh‐NC+hypoxia, and sh‐*ENO1*+hypoxia conditions. ^***^ *p* < 0.001 vs Normoxia; ^###^ *p* < 0.001 vs sh‐NC‐Hypoxia. (I) RIP‐PCR analysis showing relative *MIF* expression across input, IgG control, and anti‐ENO1 immunoprecipitations. (J) Macrophage migration assessed using Transwell assays after co‐culture with conditioned media from hypoxic SMCs treated under various conditions: control, sh‐*ENO1*, recombinant MIF (r*MIF*), and sh‐*ENO1*+r*MIF*. (K) Expression of pro‐inflammatory (*iNOS*, *CD38*, *TNF‐α*, *IL‐6*, *IL‐1β*) and anti‐inflammatory (*CD206*, *IL‐10*) markers in macrophages following co‐culture with conditioned media from hypoxic SMCs under different experimental conditions. (L) Flow cytometry quantification of CD206+CD8‐ (M2) and CD86+CD206‐ (M1) macrophage subsets following co‐culture with conditioned media from hypoxic SMCs under varied treatments. For panels A and D, differential expression analyses were meta‐analyzed using a random‐effects model; log2FC indicates integrated magnitude and direction of change, and meta‐analysis *p* values reflect pooled significance across datasets. For panels J–L, comparisons among groups were made using one‐way ANOVA followed by Tukey's post hoc test. Statistical significance is annotated as ^***^ *p* < 0.001; ##*p* < 0.01.

To define how non‐immune cells shape macrophage behaviour in AD, we systematically analysed stromal‐immune ligand‐receptor interactions. Meta‐analysis of differentially expressed ligands corresponding to macrophage‐specific receptors revealed that mesenchymal stromal cell (MSCs), fibroblasts, and vSMCs in AD secreted a common repertoire of ligands, including *SPP1*, *FN1*, *ANGPTL4*, *MIF* and *THBS2* (Figure 5A).

Among these ligands, *MIF* was markedly upregulated in MSCs, fibroblasts, and particularly in vSMCs of AD patients. Previous studies have shown that *MIF* exerts a dual role in macrophages, simultaneously restraining their migration while promoting pro‐inflammatory polarization [77, 78]. Spatial transcriptomic analysis further demonstrated the spatial proximity between vSMCs and macrophages in the aortic wall of AD samples, suggesting that vSMC‐derived signals may exert the most direct influence on macrophage function (Figure 5B). Integrating spatial context with these molecular observations, we hypothesized that vSMC‐derived *MIF* may interact with *CD74*/*CD44*/*CXCR4* receptors on macrophages, restricting their migration but amplifying pro‐inflammatory signaling, thereby fostering chronic inflammation and disease progression (Figure S8A,B). To test this hypothesis, we performed in vitro validation experiments with human vSMCs under hypoxia (1% O_2_) conditions. Cells were divided into four groups: Control, r*MIF*, sh‐NC, and sh‐*MIF*. *MIF* knockdown effectively reduced both mRNA and protein levels (Figure S8C). Conditioned medium (CM) from these groups was subsequently applied to monocyte‐derived macrophages. In the r*MIF* group, expression of *MIF*, its receptors (*CD74*, *CD44*, *CXCR4*), and M1‐associated markers (*iNOS*, *CD38*, *TNF‐α*, *IL‐6*, *IL‐1β*) was markedly increased, while M2 markers (*CD206*, *IL‐10*) were reduced (Figure S8D). These effects were reversed in the sh‐*MIF* group, consistent with flow cytometry data showing restoration of the M1/M2 macrophage ratio (Figure S8E,F). Functionally, macrophage migration was suppressed by r*MIF*‐CM but restored upon *MIF* knockdown, indicating that *MIF* limits macrophage motility (Figure S8G–I). Among the known *MIF* receptors (*CD74*, *CD44*, and *CXCR4*), we selected *CXCR4* for functional validation given the availability of a clinically used antagonist. Treatment with the *CXCR4* inhibitor AMD3100 enhanced macrophage migration and promoted M2 polarization, supporting a modulatory role for *MIF*‐receptor interactions in macrophage motility and inflammatory activation (Figure S8J–M). Collectively, these findings indicate that vSMC‐derived *MIF* impairs macrophage migration while promoting M1 polarization, driving macrophage accumulation and sustained inflammation in AD.

We next sought to delineate the upstream regulatory mechanisms governing *MIF* overexpression in vSMCs. Transcription factor prediction and meta‐analysis identified *ENO1* as a potential regulator of *MIF* expression (Figure 5C,D). Both *ENO1* and *MIF* were upregulated in AD and showed preferential enrichment and spatial colocalization within the disease‐associated SMC_C1_CP+ subtype (Figure 5E–G; Figure S8N).

These observations suggested that metabolic reprogramming characterized by *ENO1* upregulation in SMC_C1_CP+ cells may drive *MIF* overexpression in vSMCs. To verify this, human vSMCs were exposed to hypoxia (1% O_2_) under four conditions: Normoxia, Hypoxia, sh‐NC+Hypoxia, and sh‐*ENO1*+Hypoxia. *MIF* expression was robustly induced under hypoxia but was significantly attenuated upon *ENO1* knockdown (Figure 5H). RNA immunoprecipitation (RIP) confirmed that *ENO1* directly binds to MIF mRNA, indicating post‐transcriptional regulation (Figure 5I). RIP experiments further demonstrated that the MIF mRNA level was significantly higher in the anti‐*ENO1* group compared to the anti‐IgG group (Figure 5I). These results suggested that *ENO1* could regulate *MIF* expression through post‐transcriptional mechanisms.

To assess whether *ENO1*‐driven metabolic reprogramming influences macrophage behavior through *MIF* signaling, human vSMCs were cultured under hypoxic conditions (1% O_2_) and divided into four experimental groups: Control, r*MIF*, sh‐*ENO1*, and sh‐*ENO1*+r*MIF*. CM from each group were subsequently applied to macrophages for functional analyses (Figure 5J–L). r*MIF*‐CM inhibited macrophage migration, whereas sh‐*ENO1*+r*MIF*‐CM partially rescued this phenotype, suggesting that *ENO1* knockdown mitigates *MIF*‐induced migratory suppression. In parallel, macrophages exposed to r*MIF*‐CM displayed heightened M1 polarization and reduced M2 marker expression, effects reversed by *ENO1* knockdown, as confirmed by flow cytometry.

Together, these findings define a metabolic‐inflammatory axis in which *ENO1*‐driven *MIF* overexpression in disease‐associated SMC_C1_CP+ cells restrains macrophage migration and promotes their pro‐inflammatory polarization in the aortic wall, contributing to persistent inflammation and aortic dissection progression.

### 2.6 Lentivirus‐Mediated *Eno1* Knockdown Attenuates Disease Progression in AD Mouse Models via *Mif*

**FIGURE 6:**
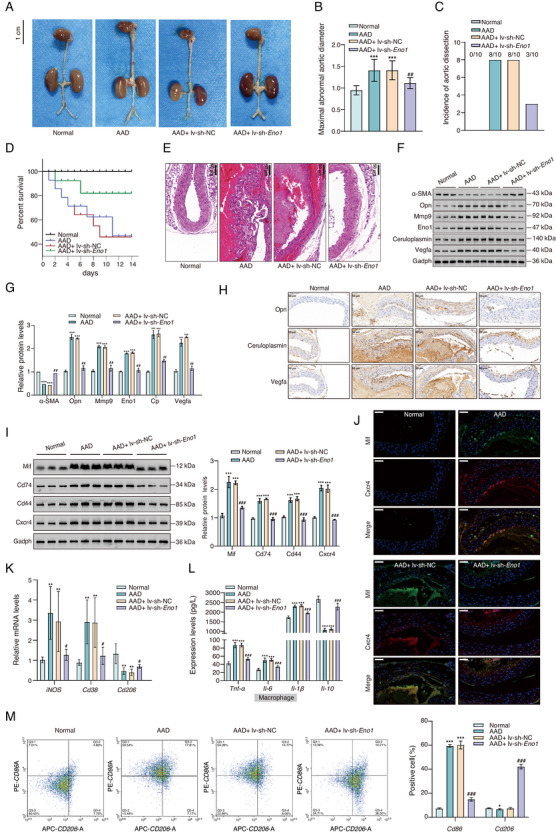
Lentivirus‐mediated *Eno1* knockdown attenuates disease progression in AAD mouse models. (A) Images showing the characteristics of aortas from four different mouse model groups: Normal (control group), AAD (BAPN+Ang‐II‐induced AAD model), AAD‐lv‐sh‐NC (empty vector transfection+Ang‐II‐induced AAD model), and AAD‐lv‐sh‐*Eno1* (*Eno1* knockdown mouse model of AAD induced by BAPN and Ang‐II). (B) Maximal abdominal aortic outer diameters in the four groups. (C) Incidence of AAD in the four groups. *n* = 10 per group. (D) Survival curves for mice in the four different mouse model groups. (E) H&E staining of aortic tissues from the four different model groups. (F,G) Western blot analysis of key proteins involved in contractile SMC (α‐SMA), synthetic SMC (Opn, Mmp9), and SMC_C1_CP+ (Ceruloplasmin, Vegfa) in aortic tissues from the four different model groups. (H) Immunohistochemistry showing synthetic smooth muscle marker Opn and SMC_C1_CP+ markers (Ceruloplasmin, Vegfa) in aortic tissues from the four different model groups. (I) Western blot analysis of Mif and its receptors in aortic tissues from the four different model groups. (J) Immunofluorescence showing Mif and Cxcr4 expression in aortic tissues from the four different model groups (Mif: green, Cxcr4: red). Scale bar = 50 µm. (K) Expression of anti‐inflammatory (*iNos*, *Cd38*) and pro‐inflammatory factors (*Cd206*) in aortic tissues from the four different model groups. (L) Expression of pro‐inflammatory (*Tnf‐α*, *Il‐6*, *Il‐1β*) and anti‐inflammatory markers (*Il‐10*) from the four different model groups. (M) Flow cytometry analysis of the ratio of Cd206+Cd86‐ M2 macrophages and Cd86+Cd206− M1 macrophages in aortic tissues from the four different model groups. For panels B, G, I, K, L and M, ANOVA followed by Tukey's post hoc test was performed for comparisons among multiple groups. ^***^ *p* < 0.001; ## *p* < 0.01.

To test the above hypothesis and explore the therapeutic potential of the *ENO1*‐*MIF* axis in AD, we established an AAD mouse model by administering BAPN combined with Ang‐II infusion. Mice were randomly assigned to four groups: Normal, AAD (model), AAD+lv‐sh‐NC (control vector), and AAD+lv‐sh‐*Eno1* (*Eno1* knockdown). Both the AAD and AAD+lv‐sh‐NC groups showed significant aortic dilation and dissection, while the AAD+lv‐sh‐*Eno1* group showed a markedly attenuated phenotype (Figure 6A). The aortic diameter in AAD and AAD+lv‐sh‐NC groups was significantly larger than controls, while the AAD+lv‐sh‐*Eno1* group showed reduced aortic dilation, lower dissection incidence and improved survival (Figure 6B–D). Histopathological changes of the aorta were consistent with these observations, as the AAD and AAD+lv‐sh‐NC groups exhibited extensive intramural hematoma, elastic fiber fragmentation and evident aortic dissection. In contrast, the AAD+lv‐sh‐*Eno1* group exhibited less severe dissection and minimal elastic fiber damage, with the aortic morphology resembling that of the control group (Figure 6E).

To evaluate the vSMC phenotype, we performed Western blot and IHC staining. In the aortas of the AAD and AAD+lv‐sh‐NC groups, the contractile marker α‐SMA was markedly downregulated, whereas the synthetic phenotype markers (Opn, Mmp9) were upregulated (Figure 6F,G). SMC_C1_CP+ markers (*Ceruloplasmin* and *Vegfa*) were significantly increased in the AAD model, indicating a phenotypic transition of vSMCs from contractile to synthetic state, especially toward the SMC_C1_CP+ subtypes (Figure 6F–H). Notably, *Eno1* knockdown markedly attenuated these phenotypic changes, suggesting its pivotal role in driving the transition toward the synthetic vSMC phenotype (Figure 6F–H). Taken together, these results indicate that the knockdown of *Eno1* expression significantly delayed the vSMC phenotype transition in the AAD mouse model, which could be one of the reasons for the reduced incidence of AAD and increased survival rate in the *Eno1* knockdown group.

Regarding macrophage inflammatory status, we next examined macrophage polarization. Compared to Normal group, both the AAD and AAD+lv‐sh‐NC groups exhibited significantly elevated levels of *Mif* and its receptors, including *Cd74*, *Cd44*, and *Cxcr4*. *Eno1* knockdown markedly reduced *Mif* expression and its receptor levels in the AAD mice, consistent with the cellular data (Figure 6I,J). Subsequently, we measured the expression of the M1 macrophage markers (*iNOS*, *Cd38*) and M2 macrophage marker (*Cd206*) in the aorta under various experimental conditions (Figure 6K,L). The results showed that the expression of M1 macrophage markers and the M1/M2 ratio were higher in the AAD and AAD+lv‐sh‐NC groups, while the expression of M2 markers was lower, and the M2 macrophage ratio was reduced. In contrast, *Eno1* knockdown reduced macrophage polarization toward the M1 phenotype in AAD mice. Furthermore, *Eno1* knockdown also suppressed the polarization of circulating macrophages toward the M1 phenotype, further highlighting the critical role of *Eno1* in regulating *Mif* and macrophage pro‐inflammatory polarization in AAD (Figure 6M). In conclusion, these findings demonstrate that the inhibition of *Eno1* expression can significantly reduce *Mif* expression, thereby attenuating pro‐inflammatory macrophage polarization in the AAD mice. This could be another reason for the reduced incidence of aortic AAD and the improved survival rate in the *Eno1* knockdown group.

## 3 Discussion

**FIGURE 7:**
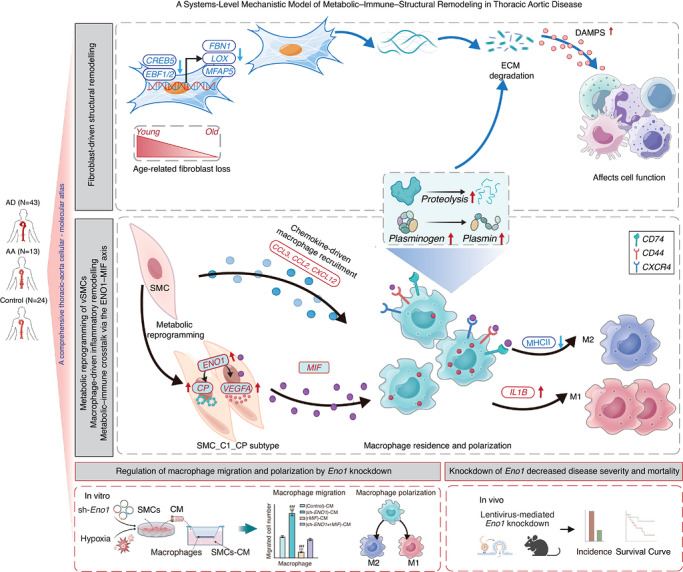
A systems‐level mechanistic model of metabolic‐immune‐structural remodeling in thoracic aortic disease.

By integrating predominantly newly generated single‐cell and spatial transcriptomic datasets, complemented by selected public resources, we established a comprehensive atlas of the human thoracic aorta (including 24 control, 13 aneurysm and 43 dissection samples). This data‐driven resource reveals extensive aortic‐wall cellular heterogeneity and coordinated stromal‐immune programs that collectively drive AD pathogenesis. As summarised in Figure 7, fibroblasts in AD exhibited marked downregulation of elastic fiber‐related genes, accompanied by expansion of a myofibroblast‐like phenotype, suggesting that impaired elastogenesis, rather than altered collagen synthesis, is the key determinant of mechanical weakening. In contrast, vSMCs underwent a glycolysis‐driven metabolic reprogramming from contractile to synthetic states. *ENO1* emerged as a pivotal regulator linking hypoxia‐induced metabolic stress to phenotypic switching, while also modulating macrophage behavior through paracrine *MIF* signaling. Concomitantly, pro‐inflammatory macrophages accumulated within the medial layer, upregulating proteolytic and fibrinolytic programs that accelerate ECM degradation. Functionally supporting this model, *ENO1* knockdown restored the contractile phenotype of vSMCs and rebalanced macrophage polarization toward an anti‐inflammatory state. In vivo, lentiviral sh‐*Eno1* treatment markedly attenuated vSMC dedifferentiation, macrophage‐driven inflammation, and disease progression, thereby identifying the *ENO1*‐*MIF* axis as a potential therapeutic target in AD. Taken together, *ENO1* emerges as a context‐responsive signaling and metabolic hub positioned at the interface of vascular remodeling and immunometabolic dysregulation. Rather than serving as a passive readout of glycolysis, *ENO1* integrates diverse upstream inputs and propagates downstream programs that converge on aberrant proliferation, inflammatory amplification, and tissue remodeling [79, 80, 81, 82, 83, 84, 85]. This expanded view of *ENO1* biology not only broadens its mechanistic relevance across human disease, but also raises the possibility that targeting *ENO1* may simultaneously modulate metabolic adaptation, inflammatory activation, and structural remodeling.

Fibroblasts, the principal ECM‐producing cells in the adventitia, have long been implicated in vascular remodeling through dysregulated ECM turnover or myofibroblast transition [86, 87, 88, 89]. Beyond confirming expansion of the myofibroblast subset in AD, our data uncovered a previously underappreciated fibroblast population, Fibro_C1_FBN1, enriched in healthy aortas and characterized by high expression of *FBN1*, *MFAP5* and *LOX*. This subtype likely supports elastic fiber assembly and ECM stability, providing a protective structural role. Strikingly, its abundance declined with aging and was nearly absent in elderly AD patients, suggesting an age‐dependent loss of elastic fiber‐maintaining fibroblasts that may underlie increased susceptibility to dissection. Given that *FBN1* is the canonical pathogenic gene in Marfan syndrome, our findings imply that downregulation of its upstream regulators (e.g., *EBF1/2*, *CREB5*) may represent a cryptic susceptibility mechanism even in the absence of coding mutations. Thus, evaluating these transcriptional regulators could complement conventional *FBN1*‐focused genetic screening and improve risk assessment in sporadic or hereditary aortic diseases.

Within the medial layer, vSMCs maintain vascular tone and integrity but exhibit remarkable plasticity in response to injury. Our single‐cell and spatial transcriptomic analyses revealed that vSMCs in AD consistently transition from a contractile to a synthetic phenotype, characterized by downregulation of *ACTA2* and upregulation of *SPP1*/*OPN*. We identified a disease‐enriched vSMC subtype, SMC_C1_CP+, displaying elevated glycolytic activity and hypoxia‐responsive signatures, including upregulation of *ENO1* and *VEGFA*. These transcriptional features reflect adaptation to chronic hypoxia and inflammation commonly observed in AD lesions [90, 91]. Mechanistically, *ENO1* serves as a metabolic switch that integrates hypoxia signaling and glycolytic flux to drive phenotypic reprogramming. Hypoxia induced *ENO1* expression alongside Ceruloplasmin and *VEGFA*, enhancing vSMC proliferation and migration, whereas *ENO1* knockdown blunted these effects both in vitro and in vivo. Collectively, these data position *ENO1* as a molecular hub that connects hypoxia‐driven metabolic remodeling to vSMC phenotypic plasticity in AD.

We further discovered that stromal cells, particularly vSMCs, robustly upregulate *MIF* in AD, a multifunctional cytokine that modulates immune cell migration and activation. Given prior reports identifying *MIF* as a non‐canonical chemokine acting via *CD74*/*CD44*/*CXCR4* [92], we explored its paracrine impact on macrophages. VSMC‐derived conditioned medium rich in *MIF* suppressed macrophage migration and promoted M1 polarization, while *ENO1* knockdown reversed these effects. Pharmacologic inhibition of *CXCR4* with AMD3100 restored macrophage motility and shifted polarization toward M2, underscoring that *MIF*‐receptor interactions modulate macrophage retention and inflammatory activation in the aortic wall. Together, these results delineate a metabolic‐immune signaling axis in which *ENO1*‐driven *MIF* expression in vSMCs impairs macrophage migration and sustains local inflammation, exacerbating ECM degradation and tissue weakening in AD. Notably, *ENO1*–*MIF* signaling is also detectable in other stromal cell populations; however, vSMCs exhibit higher expression levels and are spatially closer to macrophage‐enriched regions, suggesting a dominant contribution of vSMCs in mediating this paracrine axis.

Clinically, current AD management primarily relies on aggressive blood pressure control and surgical repair. While these strategies mitigate hemodynamic stress, they fail to directly target the cellular pathologies that drive disease progression. Our findings suggest several mechanistically informed therapeutic interventions: (i) targeting *ENO1* to disrupt hypoxia‐induced metabolic reprogramming and subsequent inflammatory signaling; (ii) modulating macrophage polarization using PPARγ agonists (e.g., rosiglitazone) to suppress M1 activation; and (iii) limiting ECM degradation via antifibrinolytic agents (e.g., aminocaproic acid). Notably, the identification of aminocaproic acid as a potential therapeutic agent highlights an additional layer of ECM regulation beyond the *ENO1*–*MIF* axis. Aminocaproic acid is a classical antifibrinolytic agent that competitively inhibits plasminogen activation and reduces the generation of plasmin, a broad‐spectrum protease capable of degrading fibrin and other matrix components. Importantly, the plasminogen–plasmin system also functions upstream of matrix metalloproteinases (MMPs), facilitating their activation and thereby amplifying proteolytic cascades within the vascular wall. In this context, plasmin‐mediated proteolysis may act as an upstream initiator, whereas macrophage‐derived MMPs represent the dominant downstream effectors driving elastic fiber fragmentation and ECM destruction. Therefore, the therapeutic rationale of aminocaproic acid lies in attenuating this upstream proteolytic amplification, which is mechanistically complementary to our core finding that fibroblast dysfunction impairs elastic fiber synthesis and that *ENO1*‐driven metabolic reprogramming promotes macrophage‐mediated inflammation. Together, these observations suggest that targeting both ECM synthesis defects and proteolytic degradation pathways may provide a more comprehensive strategy for limiting aortic wall destabilization. Such approaches could complement existing therapies and offer precisely targeted interventions aimed at halting the cellular drivers of dissection.

In summary, our comprehensive single‐cell and spatial atlas of the human thoracic aorta uncovers a multilayered remodeling network in AD, in which fibroblasts shifting toward reduced elastic support, vSMCs undergoing *ENO1*‐mediated metabolic reprogramming, and macrophages amplifying proteolytic inflammation through sustained paracrine feedback. The identification of the *ENO1*‐*MIF* axis as a bridge between metabolic stress and immune activation provides mechanistic insight into the link between genetic susceptibility and microenvironmental remodeling. This resource establishes a foundation for integrative studies of vascular pathobiology and opens new avenues for stratified risk assessment and targeted therapeutic development in aortic diseases.

The limitations of this study are also worthy of attention. First, although the BAPN + Ang II–induced AAD model is highly reproducible and effectively recapitulates key features such as medial degeneration, inflammation, and elastic fiber disruption, it primarily reflects an acute, chemically induced process driven by lysyl oxidase inhibition and hypertension, which differs from the multifactorial and chronic nature of human AD. Second, while we identify the *ENO1*–*MIF* axis as a central mechanism, the relative contributions of *ENO1* enzymatic vs. non‐enzymatic functions, as well as its interaction with hypoxia signaling pathways (e.g., *HIF‐1α*), require further investigation. Third, while candidate therapeutics were predicted and partially validated in vitro, in vivo combinatorial interventions targeting both metabolic–inflammatory signaling and ECM proteolysis remain to be explored. Future studies should aim to validate our findings in complementary models, including genetic models such as *Fbn1*‐mutant mice, and to explore targeted therapeutic strategies against the *ENO1*–*MIF* axis in more clinically representative settings.

## 4 Experimental Section

### 4.1 Ethics Declarations

All study procedures were reviewed and approved by the Ethics Committee of the People's Hospital of Xinjiang Uygur Autonomous Region, China (Approval Number: KY2023042008) and the Ethics Committee of Fudan University Shanghai Cancer Center (Approval Number: 1506147). All studies in animals were conducted in accordance with the National Institutes of Health (NIH) Guide for the Care and Use of Laboratory Animals. Written informed consent for tissue analysis and collection of clinical data was obtained from all enrolled participants or their legal guardians.

### 4.2 Study Participants Enrollment and Clinical Analysis

Participants in this study were recruited from the People's Hospital of Xinjiang Uygur Autonomous Region and diagnosed with AD (ChiCTR2300072588). Inclusion criteria adhered to the 2014 European Society of Cardiology Guidelines for aortic disease [93]. The diagnosis was confirmed using magnetic resonance imaging (MRI) or aortic CT angiography. Clinical data were systematically collected from 3403 AD patients, encompassing demographic information, blood and biochemical markers, coagulation profiles, liver and renal function tests, electrolyte levels, thyroid function tests, and metabolic parameters. Among them, 61.13% had a history of hypertension, 63.50% were overweight (BMI > 25 kg/m^2^) (Table S1). Continuous variables were filtered using an interquartile range (IQR)‐based method, with values outside Q1 − 3 x IQR to Q3 + 3 x IQR treated as outliers and set as missing. Logistic regression analyses were performed to evaluate the associations between clinical variables and disease status. Each variable was analyzed individually using generalized linear models with a binomial distribution and logit link, adjusting for age and sex where appropriate. Odds ratios (ORs) and corresponding confidence intervals (CIs) were calculated. For binary categorical variables such as sex and smoking status, significance was calculated using the Chi‐square test.

### 4.3 Tissue Preparation

Aortic tissue samples were collected from 23 AD patients who underwent aortic replacement surgery. Additionally, control samples were obtained from the aortic tissues of three organ donors who died from non‐cardiovascular causes. Tissue samples from AD patients were collected from different anatomical regions of the aortic wall, including the proximal segment (rupture site), midsegment (transition region), and distal segment (away from rupture). For single‐cell dissociation, aortic tissue (1–2 cm^2^) was dissected and cut into thin layers in Hank's Balanced Salt Solution (HBSS, without Ca^2+^ and Mg^2+^; Gibco, Waltham, MA, USA) supplemented with 10% fetal bovine serum (FBS) for further processing. For spatial transcriptome analysis, aortic tissue samples were embedded in optimal cutting temperature (OCT) compound and stored at −80°C until further use.

### 4.4 Single‐Cell RNA Sequencing (scRNA‐seq)

The mRNA capture was operated on DNBelab C4 device (MGI). Complete cDNA amplification and library construction according to the MGI DNBelab C series reagent Kit (MGI, 940‐000047‐00). In brief, the single‐cell suspensions were converted to barcoded scRNA‐seq libraries through steps including droplet encapsulation, emulsion breakage, mRNA captured bead collection, reverse transcription, cDNA amplification, and purification. cDNA production was sheared to 250–400 bp, and indexed sequencing libraries were constructed according to the manufacturer's protocol. Qualification was performed using Qubit ssDNA Assay Kit (Thermo Fisher Scientific) and Agilent Bioanalyzer 2100. All libraries were further sequenced by the DIPSEQ T1 sequencing platform generating reads containing 30‐bp read 1 (including the 10‐bp cell barcode 1, 10‐bp cell barcode 2, and 10‐bp unique molecular identifier (UMI)), 100‐bp read 2 and 10‐bp barcodes (sample index).

### 4.5 Data Preprocessing and Clustering

Raw scRNA‐seq data from our own cohort and publicly available datasets (GSE155468, GSE213740, GSE222318, GSE189795, GSE216860) were processed using R. To minimize technical artifacts, doublets were identified and removed using the DoubletFinder tool (v2.0.3) [94], and ambient RNA contamination was addressed using the decontX package [95]. Specifically, genes expressed in fewer than five cells were excluded, and cells with fewer than 800 or more than 8000 detected genes, fewer than 1000 or more than 10 000 unique molecular identifiers (UMIs), or a mitochondrial read percentage exceeding 20% were discarded. Only genes present across all datasets were retained for subsequent analysis. Gene count matrices were analyzed using the Seurat package (v4.3.0) [96]. Sample expression data were merged using the Harmony integration function to correct for batch effects and normalized using the LogNormalize function. The top 2000 highly variable genes were selected for principal component analysis (PCA), and dimensionality reduction was achieved using UMAP. Marker genes for each cell cluster were identified using the FindAllMarkers function, and cell types were assigned based on known marker genes. To further resolve the high degree of heterogeneity within the arterial tissue, subclustering was conducted following the same steps.

### 4.6 Quantification of Cell‐Type Enrichment

To assess enrichment of cell types in disease vs. control across datasets, we calculated the observed‐to‐expected ratio (Ro/e) and performed meta‐analysis based on metafor (v4.4.0) [97, 98].

### 4.7 Differential Gene Expression Analysis and Meta‐Analysis

Differential gene expression analysis was performed using the Wilcoxon rank sum test with the FindMarkers function in Seurat (v4.2.0). All genes were considered for meta‐analysis based on meta (v8.1.0) package [99]. This approach provided a weighted average of the effect sizes, giving more weight to studies with smaller standard errors, and incorporates between‐study variability to produce a more accurate and generalizable estimate of the true effect size.

### 4.8 GEPIA‐Based Pan‐Cancer Survival Analysis of Key Genes

To assess the broader prognostic pattern associated with key genes expression, disease‐free survival analysis was conducted using the GEPIA platform based on TCGA pan‐cancer datasets. All 32 tumor types available in the platform were included. Given the non‐AD nature of this dataset, the analysis was considered exploratory and was used only as an indirect reference for gene‐associated prognostic patterns.

### 4.9 Identification of Co‐Expressing Gene Modules

To better understand the complex biological systems in the study, a gene co‐expression network (GCN) analysis was conducted to associate genes with similar biological functions and facilitates comparisons across different disease stages. The R package hdWGCNA (v0.2.26), designed for high‐dimensional single‐cell RNA sequencing data, was used to perform weighted GCN analysis [100]. This analysis followed the default settings and protocol provided in the hdWGCNA tutorial (available at https://smorabit.github.io/hdWGCNA/articles/basic_tutorial.html, accessed on 20 April 2024).

### 4.10 Transcription Factor Regulatory Network Analysis

Transcription factor (TF) regulatory network analysis was performed using the SCENIC workflow (pySCENIC v0.11.2) with default parameters [101]. The SCENIC process comprises three main steps: first, co‐expression modules were inferred using GRNBoost. Second, the cisTarget database was used to identify directly regulated regulons. Third, regulon activity scores were quantified using AUCell. SCENIC was utilized to identify transcription factors that regulate different cell subtypes based on their specific gene expression profiles.

### 4.11 Cell‐Cell Interaction Analysis

In order to minimize batch effects originating from different datasets in cell‐cell communication analyses, we utilized the CellChat ligand‐receptor database to assess the average expression levels of ligands and receptors across various cell types [102]. Furthermore, we performed meta‐analysis to evaluate differential expression of these ligand‐receptor pairs under different disease states.

### 4.12 Sample Preparation and Sequencing for Spatial Enhanced Resolution Omics‐Sequencing (Stereo‐seq)

For Stereo‐seq, cryosections were sliced into 10‐µm‐thick sections. RNA quality was assessed post‐sectioning, and only sections with adequate RNA integrity (RIN > 7) were selected for further analysis. The selected tissue sections were carefully attached to the Stereo‐seq spatial transcriptomics chip. The tissue sections on the chip underwent fixation and permeabilization to release mRNA from the tissue cells. The released mRNA molecules were captured by spatially arranged capture probes on the chips, which then bound to the mRNA and reverse transcribed it into complementary DNA (cDNA). The cDNA was subsequently released from the chip, fragmented, amplified, and purified to generate a DNA nanoball (DNBs) library, following the manufacturer's protocol [103, 104]. The DNBs were then loaded onto patterned Nano arrays and sequenced on the MGI DNBSEQ‐Tx sequencer (MGI, China).

### 4.13 Stereo‐seq Data Processing and Analysis

The raw sequencing data were processed using the Stereo‐seq Analysis Workflow (SAW) software suite (https://github.com/BGIResearch/SAW), as previously described [104]. In brief, coordinate identity (CID) sequences (read 1, 1–25 bp) were matched to CID‐coordinate key‐value pairs to determine the spatial location of the tissue section (allowing for 1 mismatch). Molecular identity (MID) sequences (read 1, 26–35 bp) containing N bases or with quality scores below 10 for more than 2 bases were filtered out. cDNA sequences (read 2, 100 bp) were aligned to the reference genome (GRCm38) using STAR [105]. Expression matrices were generated for total MID counts, as well as exon and intron expression, from the processed data. For downstream analysis, we used Seurat (v4.3.0). We Initially filtered out low‐quality spots based on the following criteria: (1) at least 150 genes detected; (2) a mitochondrial read percentage of less than 30%. Additionally, genes with expression counts less than 5 were filtered out. The filtered expression data were then normalized using the SCTransform procedure.

### 4.14 Deconvolution

Spatial deconvolution analysis was conducted using the cell2location package (v.0.1.3) to estimate cell type abundance in spatial transcriptomics data [106]. Reference expression signatures for major cell types were derived from the annotated scRNA‐seq data using regularized negative binomial regressions. These signatures were then projected onto spatial transcriptome data through hierarchical Bayesian modeling to estimate the abundance of each cell type at each spot. For each spot, the cell type with the highest estimated abundance was designated as the primary cell type.

### 4.15 Spatial Transcriptomics Co‐Localization Analysis

Cell2location's results with positional information or gene expression levels were utilized as input files for MistyR (v1.2.1) to compute spatial co‐localization [107].

### 4.16 Spatial Local Moran's I Hot Spots

To evaluate the spatial distribution of gene expression, we applied the local Moran's I method using the PySAL‐esda package (available at https://github.com/pysal/esda). The local spatial autocorrelation was quantified by assessing the correlation between the expression level at a given spot (M_o_) and those of its neighboring spots (M_n_), as follows:

$$M_{o}=\left(x_{i}-\bar{x}\right)$$

$$M_{N}=\Sigma_{j=1}^{n}\;\left(x_{j}-\bar{x}\right)$$

where *n* refers to the number of nearest neighbors. In our study, we chose *n* = 8 based on the square division of each bin (bin100). The signs of *M_o_* and *M_n_* were used to classify each bin100 with a significant Moran's I coefficient into four categories: High‐High (HH), High‐Low (HL), Low‐High (LH), and Low‐Low (LL).

### 4.17 Histological Staining and Tissue Analysis

Human and mice fresh aortic tissue blocks were embedded in OCT compound and stored at −80°C until further processing. The embedded tissue samples were sectioned at a thickness of 4 µm using a Leica cryostat, and the sections were mounted onto pre‐cooled glass slides at −20°C. For hematoxylin and eosin (H&E) staining, the sections were fixed in 4% paraformaldehyde for 10 min to stabilize the cell structure. The sections were then stained with Harris hematoxylin for 5–10 min to highlight nuclei, followed by differentiation in 1% acid alcohol for 10–30 s to remove excess dye. After rinsing with running tap water, the sections were stained with eosin for 1–2 min to impart a red color to the cytoplasm. The stained sections were then dehydrated through a graded ethanol series, cleared in xylene, and mounted with glass coverslips using a non‐aqueous mounting medium to prevent drying and contamination. The tissue sections were observed under a light microscope to evaluate cellular morphology and tissue structure.

Victoria Blue staining was performed to analyze the distribution of elastic fibers in the aortic tissue samples, while Masson's trichrome staining (SH012; CNT‐BIO) was used to detect collagen fibers. The sections were assessed microscopically for the presence and organization of elastic and collagen fibers to provide insights into the structural integrity of the aortic tissue.

### 4.18 Cell Cultivation

Human aortic smooth muscle cells (HASMCs, CP‐H081) and human myeloid leukemia cell line *THP‐1* (CL‐0233) were obtained from Procell (Wuhan, China). HASMCs were cultured in complete HASMC medium (CM‐H081, Procell). *THP‐1* cells were cultured in *THP‐1* cell‐specific medium (CM‐0233, Procell) containing 10% FBS (164210‐50, Procell), 0.05 mm β‐mercaptoethanol (PB180633, Procell), and 1% Penicillin‐Streptomycin (PB180120, Procell). All cell types were cultivated at 37°C in a humidified atmosphere containing 5% CO_2_.

### 4.19 Cell Transfection and Treatment

To achieve knockdown of *ENO1* or *MIF*, SMCs were transfected with *ENO1* or *MIF*‐targeted short hairpin RNA (sh‐*ENO1* and sh‐*MIF*), with sh‐NC serving as a negative control. Transfection was performed using OriTransPEI‐DNA (ORI2305, Ori‐Bio, Changsha, China) according to the manufacturer's protocol. After 48 h, cells were harvested for RNA, protein, or co‐culture experiments. Transfection efficiency was validated by qRT‐PCR and Western blotting (see Table S3 for siRNA and plasmid sequences). For hypoxia treatment, SMCs were cultured in a hypoxia chamber (94%N_2_, 5%CO_2_, and 1%O_2_) for 48 h. Control SMCs were cultured under normoxic conditions (5%CO_2_ and 20%O_2_) at 37°C for 48 h. After treatment, media were collected, centrifuged to remove cell debris, and stored at ‐80°C until use.

*THP‐1* human monocytic leukemia cells were differentiated into macrophages using 20 ng/mL M‐CSF for 7 days. *THP‐1* macrophages were then co‐cultured with CM from transfected and treated SMCs and divided into six groups (n = 3 for each group): (control)‐CM, (r*MIF*)‐CM, (sh‐NC)‐CM, (sh‐*MIF*)‐CM. (sh‐*ENO1*)‐CM, and (sh‐*ENO1*+ r*MIF*)‐CM. Functional responses of treated *THP‐1* macrophages were assessed. In some experiments, *THP‐1* macrophages were treated with 2 µg/mL *CXCR4* antagonist AMD3100 (HY‐10046, MedChem Express, Monmouth Junction, USA), 10 ng/ml recombinant human *MIF* protein (r*MIF*; 10246‐H08H, Sino Biological, Beijing, China), or vehicles for 24 h before functional evaluations.

### 4.20 Macrophage Migration Assay

Macrophage migration was assessed using a Transwell assay with an 8 µm pore size insert (Corning). Macrophages (1 × 10^5^ cells) were seeded in the upper chamber in serum‐free media, while conditioned media from SMCs was placed in the lower chamber. After incubation for 24 h at 37°C, non‐migrated cells on the upper side of the insert membrane were gently removed with a cotton swab, and the migrated cells on the lower membrane were fixed with 4% paraformaldehyde, stained with 0.1% crystal violet, and counted in five randomly selected fields under a light microscope. Data were presented as the average number of migrated cells per field.

### 4.21 Macrophage Polarization Assay

Macrophage polarization was assessed by flow cytometry. After 48 h of co‐culture with SMC‐conditioned media, macrophages were harvested and stained with antibodies against CD86 (M1 marker; 374206, BioLegend, San Diego, USA) and CD206 (M2 marker; E‐AB‐F1161E, Elabscience, Wuhan, China). The stained cells were analyzed using a BD FACSCanto II flow cytometer, and data were processed using FlowJo software. The proportions of M1 and M2 macrophages were determined based on *CD86*+ and *CD206*+ populations, respectively.

### 4.22 qRT‐PCR Analysis

Primers for the target genes (*ENO1*, *MIF*, *CD74*, *CD44*, *CXCR4*, *iNOS*, *CD38*, *CD206*, *Arg1*, and *GAPDH*) were designed using NCBI Primer‐BLAST. Total RNA was extracted from cells using TRIzol reagent, and cDNA was synthesized using the RevertAid First Strand cDNA Synthesis Kit (Thermo Fisher). qRT‐PCR was performed with PowerUp SYBR Green Master Mix on an ABI 7500 system. Relative expression levels were calculated using the 2−ΔΔCt method, normalized to GAPDH. Primer sequences are listed in Table S6.

### 4.23 Immunoblotting

Total protein was extracted from SMCs and aortic tissues using radioimmunoprecipitation assay (RIPA) buffer (Beyotime, China). Protein concentrations were measured using a bicinchoninic acid (BCA) protein assay kit (Thermo Fisher Scientific, USA). Equal amounts of protein (40 µg per sample) were separated on a 10% SDS‐PAGE gel and transferred to polyvinylidene difluoride (PVDF) membranes (Millipore, USA). Membranes were blocked for 1 h at room temperature with 5% bovine serum albumin (BSA; Sigma‐Aldrich) in Tris‐buffered saline containing 0.1% Tween‐20 (TBST). Following blocking, membranes were incubated overnight at 4°C with primary antibodies diluted in TBST: MT‐CO2 (1:2000, 55070‐1‐AP, Wuhan, China), COXIV (1:2000, 11242‐1‐AP, Proteintech), MCT1 (1:2000, 20139‐1‐AP, Proteintech), MCT4 (1:1000, ab308528, Abcam, Cambridge, UK), PKM2 (1:2000, 15822‐1‐AP, Proteintech), ENO1 (1:1000, ab227978, Abcam), α‐SMA (1:1000, 14395‐1‐AP, Proteintech), OPN (1:1000, 22952‐1‐AP, Proteintech), MMP9 (1:500, 10375‐2‐AP, Proteintech), Ceruloplasmin (1:1000, 66156‐1‐Ig, Proteintech), MIF (1:1000, ab227073, Abcam), VEGFA (1:2000, 19003‐1‐AP, Proteintech), iNOS (1:1000, ab178945, Abcam), CD38 (1:1000, ab108403, Abcam), CD206 (1:5000, 81525‐1‐RR, Proteintech), Arg1 (1:5000, 16001‐1‐AP, Proteintech), and GAPDH (1:3000, AF7021, Affinity, Cincinnati, OH, USA). After washing, the membranes were incubated for 1 h at room temperature with horseradish peroxidase (HRP)‐conjugated secondary antibodies (goat anti‐rabbit IgG, 1:5000, ab6721, Abcam) in TBST. Protein bands were visualized using enhanced chemiluminescence (ECL) reagents (Thermo Fisher Scientific) and detected using an ImageQuant LAS 4000 system (GE Healthcare). Quantification of band intensity was performed using ImageJ software (NIH).

### 4.24 Immunohistochemical Staining

For IHC staining, human and mice aortic tissue sections were fixed in 4% paraformaldehyde, embedded in paraffin, and cut into 4 µm sections. The sections were deparaffinized in xylene and rehydrated through a graded ethanol series. Antigen retrieval was performed by boiling the sections in citrate buffer (pH 6.0) for 20 min. After cooling, the sections were blocked with 5% BSA for 30 min at room temperature. Primary antibodies against ENO1 (1:2000, ab227978, Abcam), MIF (1:500, ab227073, Abcam), OPN (1:500, 22952‐1‐AP, Proteintech), Ceruloplasmin (1:200, 66156‐1‐Ig, Proteintech), and VEGFA (1:100, 19003‐1‐AP, Proteintech) were applied overnight at 4°C. The next day, sections were washed with phosphate‐buffered saline (PBS) and incubated for 1 h at room temperature with HRP‐conjugated secondary antibodies (goat anti‐rabbit IgG, 1:500, ab6721, Abcam). The antigen‐antibody complex was visualized using diaminobenzidine (DAB) substrate (Vector Laboratories), and counterstaining was performed with hematoxylin. The stained sections were imaged using a light microscope, and quantitative analysis of staining intensity and distribution was conducted using ImageJ software.

### 4.25 ATP Synthesis and Lactate Production Assays

To evaluate ATP synthesis and lactate production, commercial assay kits were used according to the manufacturer's instructions. SMCs were seeded in 6‐well plates at a density of 2 × 10^5^ cells per well and cultured under normoxic (20% O_2_) or hypoxic (1% O_2_) conditions for 24 or 48 h. For ATP measurements, cell lysates were prepared, and ATP content was quantified using a luciferase‐based luminescence assay. For lactate measurements, the culture supernatants were collected, and lactate concentration was measured spectrophotometrically at 570 nm using the lactate assay kit. Results were normalized to total protein content. ATP content assay kit (A095‐1‐1, Jiancheng Bioengineering Institute, Nanjing, China) and L‐lactate assay kit (A019‐2‐1, Jiancheng Bioengineering Institute) were used.

### 4.26 Glucose Consumption Assay

Glucose consumption was measured using the Glucose Assay Kit (A154‐1‐1, Jiancheng Bioengineering Institute). SMCs were seeded in 6‐well plates and cultured in glucose‐free DMEM under hypoxic (1% O_2_) or normoxic (20% O_2_) conditions for 24–48 h. The glucose concentration in the supernatant was measured by detecting glucose oxidation, which is coupled to the generation of a colorimetric product at 450 nm. Glucose consumption was calculated by subtracting the residual glucose concentration from the initial glucose concentration.

### 4.27 MitoSOX Red Staining

SMCs were cultured under hypoxic (1% O_2_) or normoxic (20% O_2_) conditions for 24 h. Mitochondrial ROS levels were measured using the MitoSOX Red Mitochondrial Superoxide Indicator (Thermo Fisher). Cells were incubated with 5 µm MitoSOX Red for 10 min at 37°C, followed by washing with PBS. ROS levels were analyzed using flow cytometry (BD FACSCanto II) or visualized under a fluorescence microscope (excitation/emission: 510/580 nm).

### 4.28 JC‐1 Staining for Mitochondrial Membrane Potential

To assess mitochondrial membrane potential, cells were stained with the JC‐1 Mitochondrial Membrane Potential Detection Kit (Abcam). Cells were incubated with 5 µm JC‐1 dye for 20 min at 37°C, then washed with PBS. Analysis was performed using flow cytometry or fluorescence microscopy. Depolarized mitochondria were indicated by a shift from red to green fluorescence (excitation/emission: 585/510 nm).

### 4.29 ELISA Assay

To assess the levels of inflammatory cytokines, including *TNF‐α*, *IL‐6*, *IL‐1β*, and *IL‐10*, ELISA was performed using commercially available kits. Mouse serum samples or supernatants from media of macrophages co‐cultured with SMC‐conditioned media were collected and analyzed. ELISA kits for TNF‐α (CSB‐E04740h, Cusabio, Wuhan, China), IL‐6 (CSB‐E04638h, Cusabio), IL‐1β (CSB‐E08053h, Cusabio), and IL‐10 (CSB‐E04593h, Cusabio) were used according to the manufacturer's instructions. Absorbance was measured at 450 nm using a microplate reader (Thermo Fisher Scientific, USA). A standard curve was generated for each cytokine, and cytokine concentrations were calculated based on the standard curve. All samples were run in triplicate, and data were normalized to the control group.

### 4.30 Scratch Wound Healing Assay

Macrophages were seeded into 6‐well plates and cultured until reaching 90% confluency. A uniform linear scratch was created across the macrophage monolayer using a sterile 200 µL pipette tip. Detached cells were removed by washing the wells with PBS. Macrophages were then cultured in serum‐free RPMI‐1640 medium supplemented with conditioned media from SMCs with different treatments and incubated under hypoxic (1% O_2_) conditions. The conditioned media groups included control, recombinant MIF or THBS2, sh‐YBX3, sh‐ENO1, or combinations of these treatments. Images of the scratch area were taken at 0 and 24 h using a phase‐contrast microscope. The width of the remaining wound gap was measured using ImageJ software, and the percentage of wound closure was calculated.

### 4.31 Flow Cytometry Analysis

After transfection, cells were harvested, fixed in 4% paraformaldehyde, and permeabilized with 0.1% Triton X‐100. The cells were then incubated with primary antibodies against YBX3 or ENO1, followed by secondary antibodies conjugated with FITC. Fluorescence was measured using a flow cytometer (BD FACSCanto II), and the knockdown efficiency was quantified as a reduction in mean fluorescence intensity (MFI) compared to controls.

### 4.32 RNA Immunoprecipitation (RIP)

SMCs were lysed, and the lysates were incubated with anti‐*ENO1* antibody or control IgG antibody overnight at 4°C. Protein‐RNA complexes were precipitated using Protein A/G agarose beads. RNA was extracted from the precipitated complexes using TRIzol, and *MIF* mRNA levels were quantified by qRT‐PCR. The fold enrichment of *MIF* mRNA in the *ENO1*‐immunoprecipitated (IP) group compared to the IgG control group was calculated.

### 4.33 Mouse Aortic Dissection Model

A total of 33 wildtype four‐week‐old male C57 mice were obtained from Hunan SJA Laboratory Animal Co., Ltd. (Changsha, China). The study protocol of using animals for research was approved by the Medical Ethics Committee of People's Hospital of Xinjiang Uygur Autonomous Region, China (Approval Number: KY2023042008). Mice were housed in an environment with controlled temperature and humidity under specific‐pathogen‐free conditions and fed sterile food and water.

Thirty‐three mice were randomly allocated into four groups: Normal (n = 6), AAD (n = 9), AAD+ lv‐sh‐NC (n = 9), and AAD+ lv‐sh‐*Eno1* (n = 9). Normal mice were given free feeding. Mice from the latter 2 groups were subjected to intravenous injection with 100 µl of lv‐sh‐NC or lv‐sh‐*Eno1* lentivirus (1 × 10^7^ TU) via tail vein twice (at day 0, 8). Mice from the latter 3 groups were fed a normal diet and administered freshly prepared BAPN (Sigma‐Aldrich, St. Louis, USA) solution dissolved in the drinking water (0.1 g/kg/d) for 16 days. The drinking water was changed twice a week, the amount of drinking water was recorded, and the weight of the mice was weighed before changing the water. At the same time, three groups of model mice were subcutaneously injected with Ang‐II (the concentration was adjusted according to time): Ang‐II dose was 1.5 mg/kg/d on days 1–7, Ang‐II was 0.75 mg/kg/d on days 8–14 days, and Ang‐II was 0.375 mg/kg/d on days 15–16 days. The normal group was injected with an equal volume of normal saline. All mice that died before the expected end time of the experiment were autopsied immediately, and blood clots were found in the thoracic cavities of these mice. Mice surviving at the end of the experiment were sacrificed by an overdose of sodium pentobarbital, and their blood and tissue samples were collected for further analyses.

### 4.34 Proteome Sample Preparation

The OCT‐embedded cryosections were initially rinsed with pre‐cooled PBS to remove the OCT, after which the tissue samples were transferred into centrifuge tubes for protein extraction. The samples were lysed in a TCEP buffer containing 2% sodium deoxycholate, 40 mm 2‐chloroacetamide, 100 mm Tris‐HCl, and 10 mm Tris (2‐carboxyethyl) phosphine at pH 8.5, along with protease inhibitors, at 99°C for 30 min. After cooling to room temperature, trypsin (Promega, Madison, WI, USA, #V5280) was introduced, and digestion was carried out for 16 h at 37°C. Following this, 10% formic acid (FA) was added, and the mixture was vortexed for 3 min before centrifugation at 12 000 g for 5 min. Subsequently, a new 1.5 mL tube containing extraction buffer (0.1% formic acid in 50% acetonitrile) was utilized to extract the supernatant, which was vortexed for 3 min and then centrifuged at 12 000 g for another 5 min. The collected supernatant was transferred to a fresh tube and dried using a speed‐vac at 60°C. Then, 0.1% formic acid was added to resuspend the dried peptides, followed by vortexing for 3 min and centrifugation at 12 000 g for 5 min to separate the supernatant. The supernatant was then desalted using a column packed with two layers of octadecyl (C18) (Empore, Lot #3M‐2215). The resulting peptides were evaporated to dryness using a Speed‐Vac at 60°C. Subsequently, the peptides were resuspended in 0.1% FA, and their concentration was measured via a NanoDrop ND‐2000 spectrophotometer.

In addition to the processing of individual samples, aliquots were pooled from each sample to create a peptide mixture for high‐pH reversed‐phase fractionation, which was used to generate a matching spectral library.

### 4.35 High‐pH Reversed‐Phase Fractionation

Before fractionation, the peptide mixture was dried under vacuum and reconstituted in 0.1% FA. Peptides were fractionated using a 2.1 mm × 150 mm ACQUITY UPLC Peptide BEH C18 column with 1.7 µm beads (Waters). The separation was performed on a Waters ACQUITY UPLC H‐Class Bio instrument via high‐pH reversed‐phase liquid chromatography (RPLC) with solvent A (20% acetonitrile + 0.4% ammonia) and a non‐linear increasing concentration of solvent B (80% acetonitrile + 0.08% ammonia) at a flow rate of 0.3 mL/min. The 60 min separation gradient was set as follows: 2%–4% B in 10 min; 4–8% B in 2 min; 8%–30% B in 36 min; 30%–70% B in 2 min; 70%–100% B in 1 min; and 100% B in 9 min. Peptides were separated and collected every minute, resulting in a total of 45 fractions from 9 min to 55 min, which were then combined into 15 fractions using a stepwise concatenation strategy. Subsequently, the fractions were dried in a Speed‐Vac and re‐dissolved in 0.1% FA for subsequent analysis.

### 4.36 Liquid Chromatography and Mass Spectrometry (LC‐MS/MS)

Nanoflow LC‐MS/MS analysis of peptides was conducted on a TimsTOF Pro 2 mass spectrometer (Bruker) coupled to a nanoElute nanoHPLC system. 500  ng of peptides were loaded on a 2‐cm self‐packed trap column (100 µm inner diameter, 3 µm ReproSil‐Pur C18‐AQ beads, Dr Maisch GmbH) using Solvent A and separated in an in‐house packed C18 analytical column (20 cm × 150 µm ID, ReproSil‐Pur 120 C18‐AQ, 1.9 µm, Dr. Maisch GmbH). Peptides were eluted using a 75 min gradient (Solvent A: 0.1% FA in water; Solvent B: 0.1% FA in 80% ACN) at a constant flow rate of 600 nL/min (0–75 min, 0 min, 4% B; 0–15 min, 4%‐12% B; 15–65 min, 12%‐24% B; 65–72 min, 24%–40% B; 72–75 min, 40%‐80%). Column temperature was kept at 60°C using a column heater. After separation by chromatography, the samples were analyzed using a timsTOF Pro 2 mass spectrometer.

For sample analysis, data were acquired using the diaPASEF method [108]. The acquisition scheme comprised 8 cycles, which included 21 mass steps (with m/z ranging from 475 to 1000 and a mass width of 25 Da) and covered an ion mobility range from 0.85 to 1.27 V·s/cm^2^. The TIMS‐MS survey scan was set to 100–1700 m/z and 1/K_0_ 0.85–1.30 V·s/cm^2^. Each PASEF scan had an acquisition time of 100 ms, yielding a near 100% duty cycle and a total cycle time of around 0.95 s. The collision energy was ramped based on ion mobility, from 20 eV at 1/K_0_ = 0.6 V·s/cm^2^ to 59 eV at 1/K_0_ = 1.60 V·s/cm^2^.

For spectral library generation, factions were measured in data‐dependent acquisition (DDA) model with PASEF [109]. The number of PASEF was four and total cycle time was 0.53 s. Singly charged precursors were excluded by their position in the m/z‐ion mobility plane and precursors that reached a “target value” of 20,000 a.u. were dynamically excluded for 0.4  min. We used 100  ms to accumulate and elute ions in the TIMS tunnel. The MS1 m/z‐range was acquired from 100 to 1700, and the ion mobility range from 1.3 to 0.85  V.s/cm^2^.

### 4.37 MS Data Analysis

For the construction of spectral libraries, we engaged the FragPipe computational platform (version 21.1), equipped with MSFragger (version 4.0), Philosopher (version 5.1.0), and Python (version 3.10.9) [110, 111]. The ddaPASEF raw files of fractions, throughout the spectral library construction, were cohesively processed. MSFragger was used to search files against the UniProt human protein database (reviewed sequences only; as updated on 2024.02.26, housing 20 423 entries), which also encompassed reversed protein sequences appended as decoys for ensuing false discovery rate (FDR) calculations. The mass tolerances were: 20 ppm for precursor and 0.05 Da for product ions, enabling spectrum deisotoping, mass calibration, and parameter optimization. We established enzyme specificity to “trypsin” and permitted up to 2 missed trypsin cleavages. Configurations for peptide length ranged between 7 and 50, while peptide mass was set from 500 to 5000 Da. Cysteine carbamidomethylation was fixed as a modification, while variable modifications entailed N‐acetylation and methionine oxidation. The resulting peptide‐spectrum matches were adjusted to a 1% false discovery rate and converted to a spectra library contained 592 151 psms, representing 7622 proteins.

diaPASEF raw files were searched against the generated spectra library using DIA‐NN (version 1.8.1) [112]. Analysis was performed using default parameters in the “robust LC (high precision)” mode with RT‐dependent median‐based cross‐run normalization.

Protein quantification was performed using the MaxLFQ algorithm as implemented in the iq R‐package (https://github.com/vdemichev/ diann‐rpackage, version 1.0, commit “eb4607a”) according to the common data procession [113].

### 4.38 Statistical Analysis

Cell experiments were performed at least three times, while animal experiments were conducted at least six times. For the aortic dissection studies, a minimum of six mice per group was required to detect a significant mean difference at 16 days; thus, each group was assigned nine to ten mice. The statistical analyses were performed using R package (version 4.1.0; https://www.r‐project.org, accessed on 20 August 2022), GraphPad Prism 8.0 software (GraphPad Software, San Diego, CA, USA), and SPSS software (version 20.0; Chicago, IL, USA). Continuous variables are presented as the mean ± standard deviation (SD), while categorical variables are presented as counts or percentage (%). Comparisons between two groups were analyzed using Student's t test, and comparisons among multi‐groups were analyzed using one‐way analysis of variance (ANOVA) followed by Tukey's post hoc test. A false discovery rate (FDR) of < 0.05 was considered statistically significant.

## Author Contributions

J. T., H. Y., J. Y., and X. C. contributed equally to this work. J. T., H. Y., and J. S. drafted the original and revised manuscript. Conceptualization was performed by J. T., J. S., X. F., and Y. Y. Methodology was developed by J. T., and Y. Y. Data curation were carried out by J. Y., D. L., and J. W. Investigation were performed by J. T., J. Y., Q. Z., L. Y., L. P., F. L., S. P., F. Y., H. Z., and Z. W. Validation were performed by Q. Z., L. Y., F. L., J. S., and X. F. Formal analysis were conducted by H. Y., J. Y., X. C., X. W., M. Y., D. L., and J. S. Supervision and funding acquisition were carried out by H. P., and Y. Y. Visualization was performed by H. Y. and J. S. Project administration were provided by R. C., Z. D., Y. W., Y. C., Z. Y., H. P., and Y. Y. All authors participated in reviewing and editing the manuscript, and approved the final version for publication.

## Conflicts of Interest

The authors declare no conflict of interest.

## Supporting information




**Supporting File 1**: advs75509‐sup‐0001‐SuppMat.docx.

**Supporting File 2**: advs75509‐sup‐0002‐Tables S1‐S6.xlsx. [Correction added on 12 May 2026 after first online publication: Updated Supporting Information file is available in this version.]

## Data Availability

The scRNA‐seq of this study supporting the findings of this study have been deposited into CNSA (CNGB Sequence Archive) of CNGBdb (https://db.cngb.org/cnsa/) under the accession number CNP0007139 and Stereo‐seq data have been deposited into STOmisDB with accession number STT0000166 [114]. The data that support the findings of this study are available on request from the corresponding author.
