# Supplementary material for: Integrated Single‐Cell and Spatial Analysis Reveals a Metabolic‐Immune Axis Driving Aortic Dissection
Source: Adv Sci (Weinh). 2026 May 10;13(41):e75509. doi: 10.1002/advs.75509 (PMC13335668; doi:10.1002/advs.75509)
Supplement: Supplementary file 1 — Supporting File 1: advs75509‐sup‐0001‐SuppMat.docx. [file ADVS-13-e75509-s002.docx]

**Integrated Single-Cell and Spatial Analysis Reveals A Metabolic–Immune Axis Driving Aortic Dissection**

Jing Tao, Huanjie Yang, Jiahui Yong, Xueting Chen, Qiang Zhao, Xueli Wu, Lei Yan, Li Pang, Fan Luo, Mengjun Yu, Shanshan Pan, Deyang Li, Rouxi Chen, Juan Wang, Zhensheng Dong, Fan Yang, Yue Wang, Yang Chen, Hongjian Zheng, Zhimin Yang, Zijie Wang, Karsten Kristiansen, Hui Peng*, Xiaodong Fang*, Juan Shen* and Yining Yang*

**Supplemental Figures**

**
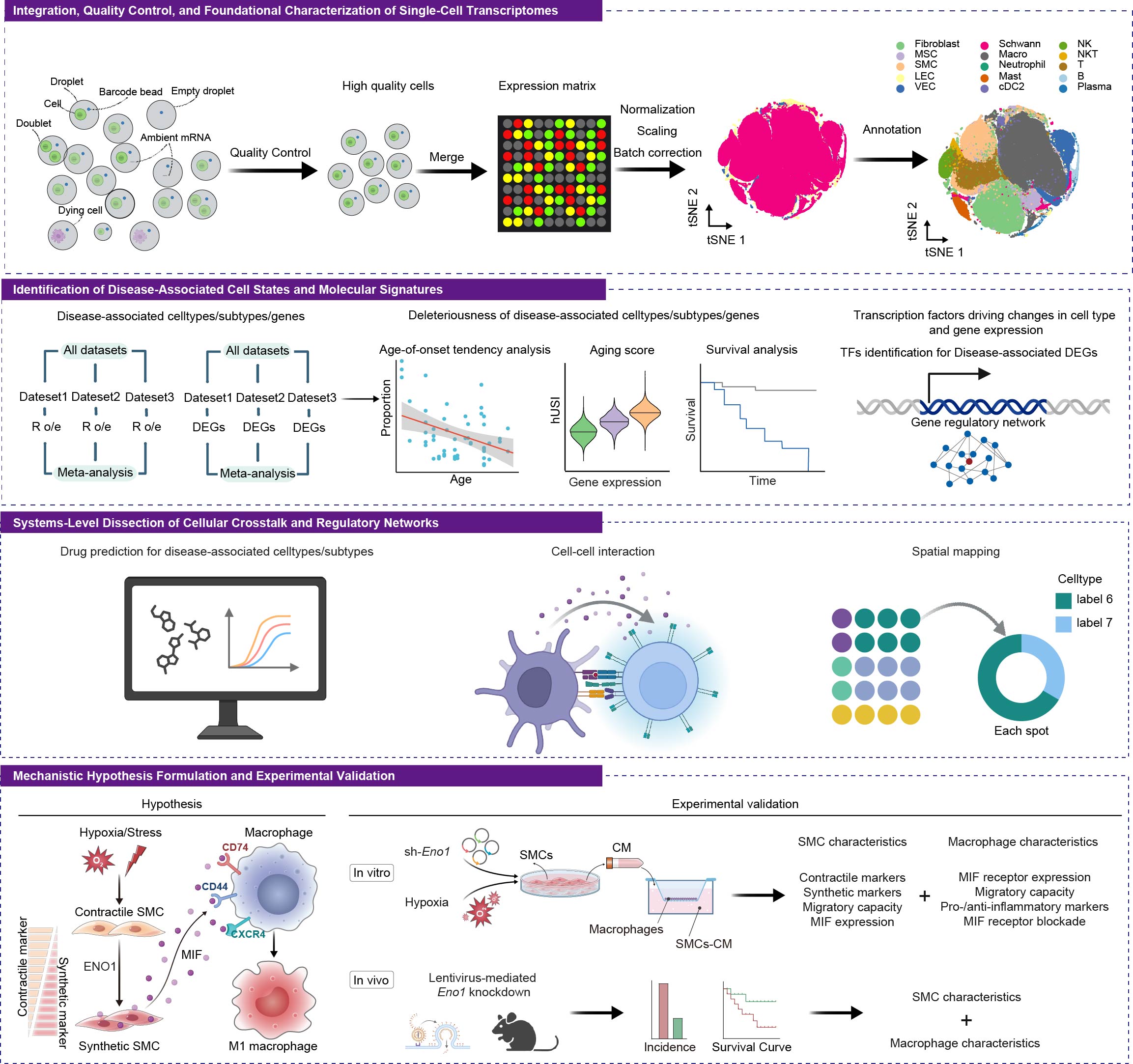
**

**Figure S1.** Integration and analysis workflow of multiple datasets.

**
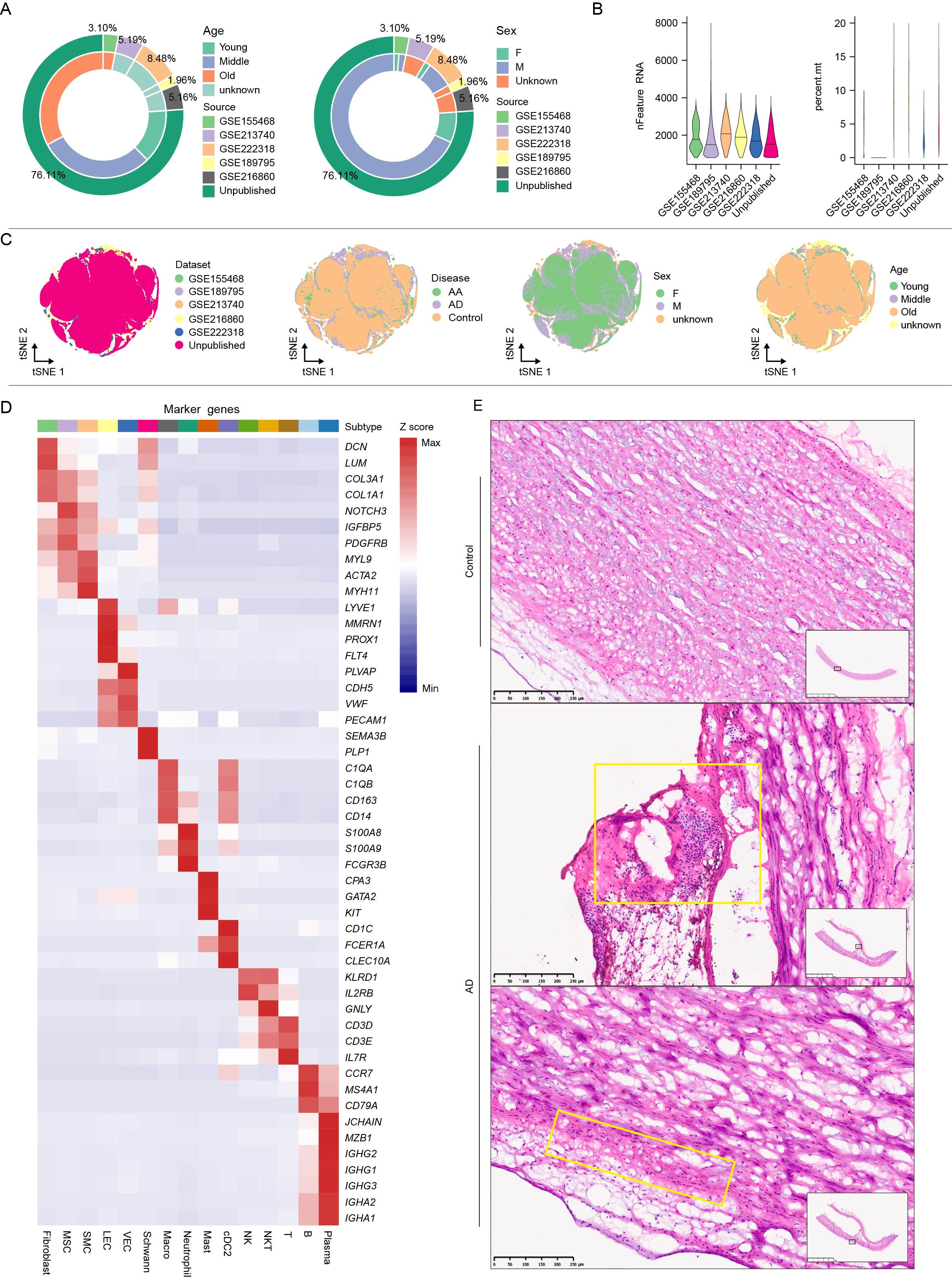
**

**Figure S2.** Baseline demographics and single-cell dataset overview across disease conditions. A) Proportions of cells stratified by age and sex across datasets under different disease states. B) Violin plots showing per-cell gene count (n_genes) and mitochondrial content percentage across samples. C) t-SNE visualization of single-cell data clustered by dataset, disease condition, gender, and age group. D) Heatmap of canonical marker gene Z-score across annotated cell types. E) Representative hematoxylin and eosin (H&E) stained images of ascending aortic tissues from different disease states.


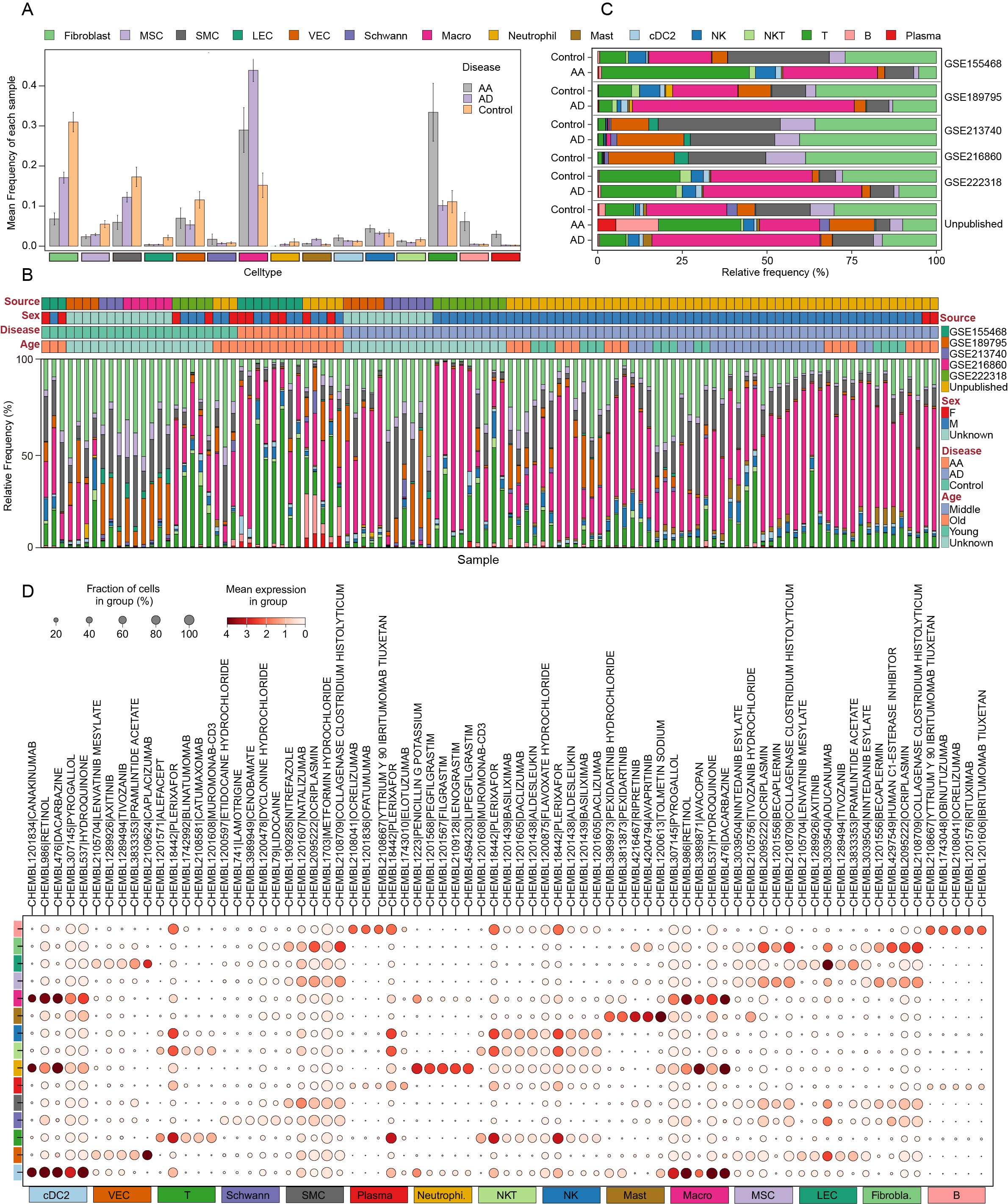


**Figure S3.** Cellular composition across samples and drug-target mapping across celltypes. A) Bar plots showing the frequency distribution of major cell types across disease states. B) Stacked bar charts depicting the proportional composition of major cell types in each sample; detailed clinical attributes are displayed alongside. C) Stacked bar plots showing relative cell-type frequencies across different datasets. D) Matrix plot visualizing cell-type-specific drug targets inferred using drug2cell.


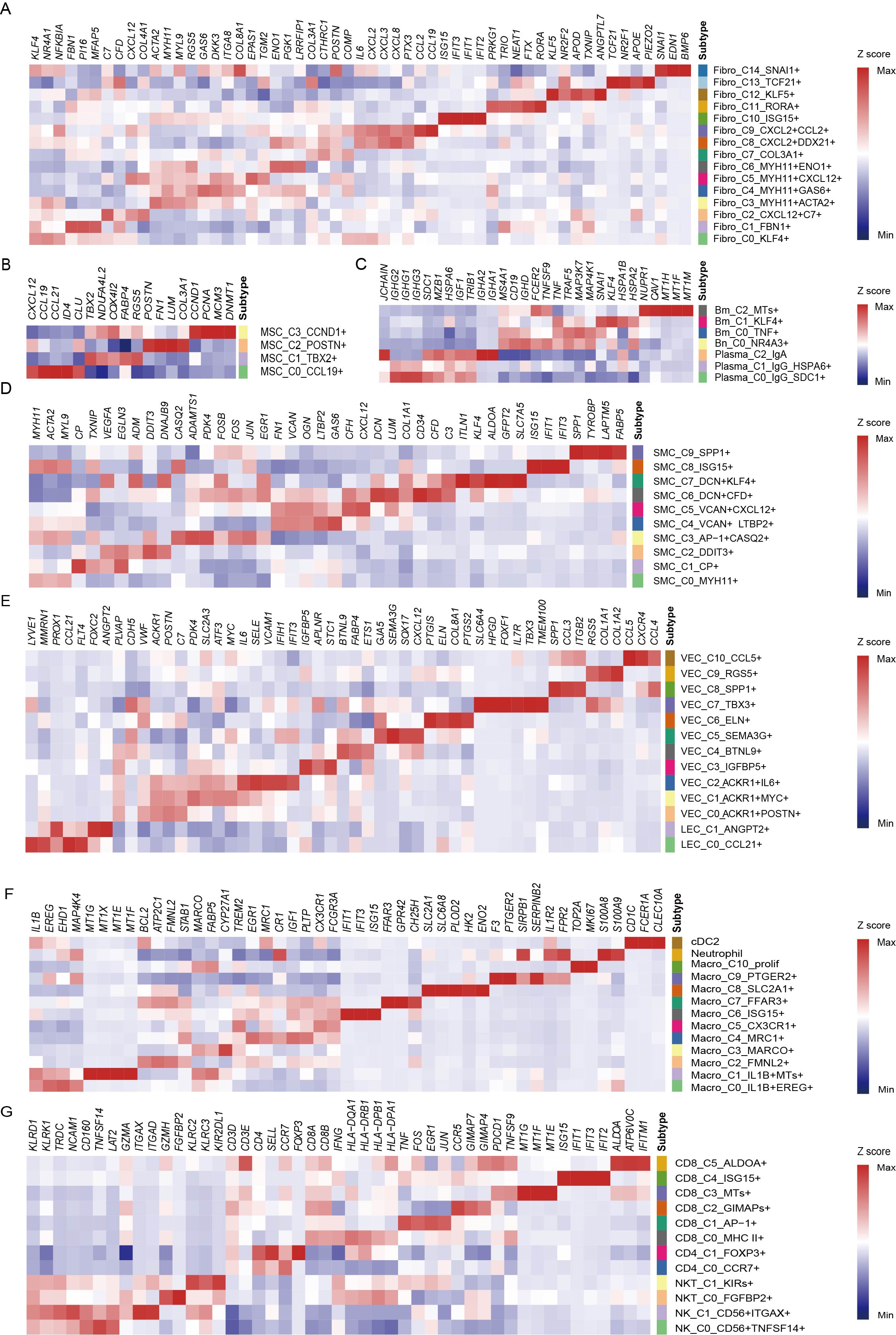


**Figure S4.** Subclassification of non-immune cell types based on marker gene Z-score. A) Heatmap of marker gene Z-score defining fibroblast subtypes. B) Heatmap of marker gene Z-score defining MSC subtypes. C) Heatmap of marker gene Z-score defining subtypes within B cells and plasma cells. D) Heatmap of marker gene Z-score defining vSMC subtypes. E) Heatmap of marker gene Z-score defining EC subtypes. F) Heatmap of marker gene Z-score defining subtypes within myeloid lineage cells. G) Heatmap of marker gene Z-score defining subtypes within T, NK, and NKT cells.

**
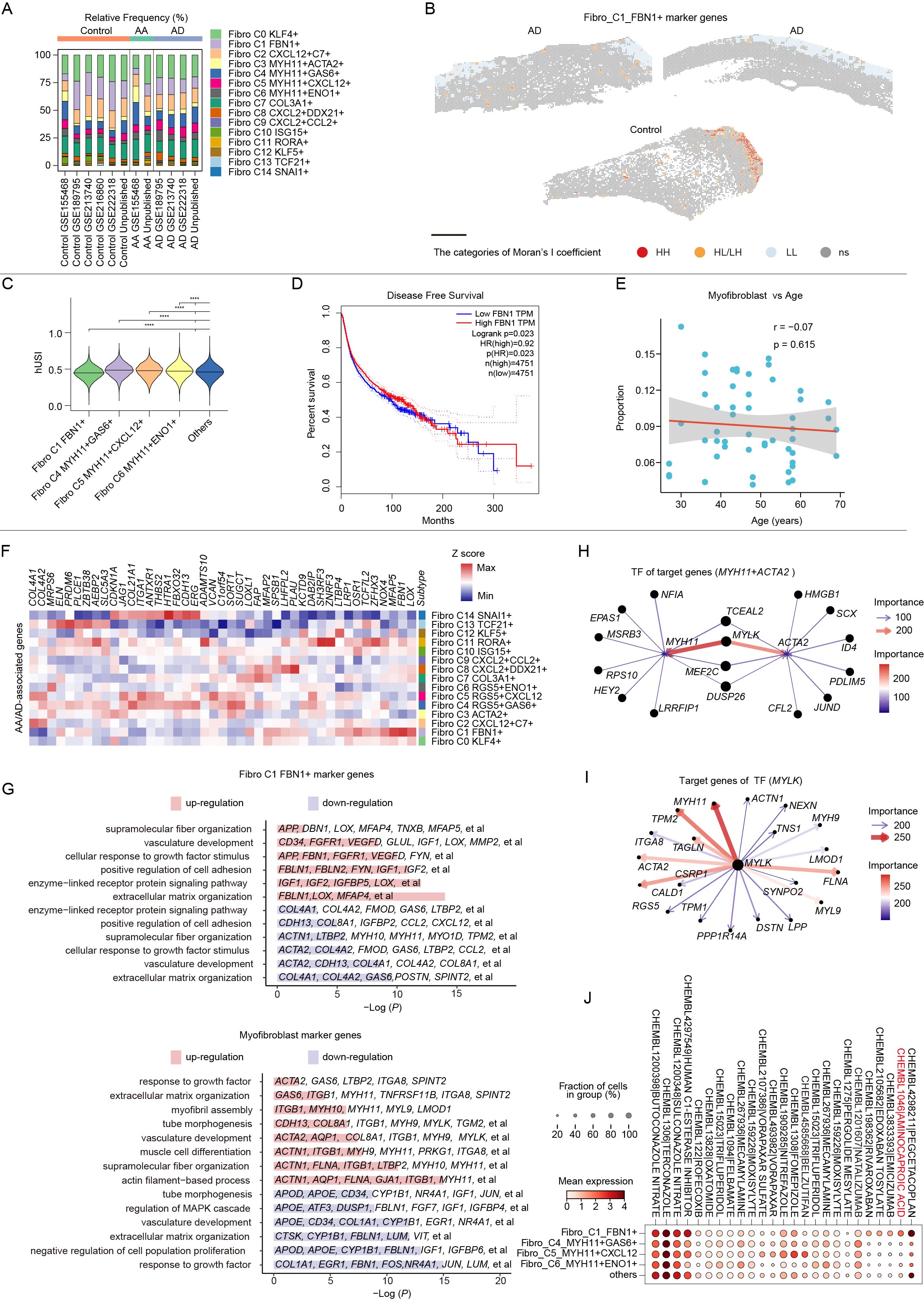
**

**Figure S5.** Characterization of fibroblast subtypes across disease conditions. A) Stacked bar plots showing the relative frequencies of fibroblast subtypes across disease states. B) Hotspot localization via Local Moran’s I for Fibro_C1_FBN1+ marker genes (*FBN1*, *MFAP5*, *ELN*) across all human aortic tissues. Scale bars = 2 mm. C) hUSI scores across fibroblast subtypes in the unpublished dataset, compared with the “Others” group using Wilcoxon rank-sum tests. Significance levels are indicated as *P < 0.05, **P < 0.01, ***P < 0.001, and ****P < 0.0001. D) Disease-free survival analysis of *FBN1* expression based on the TCGA pan-cancer cohort using the GEPIA platform (http://gepia.cancer-pku.cn/detail.php). All 32 cancer types available in the platform were included. This analysis is presented only as a general reference for prognostic gene-expression patterns and should not be interpreted as a direct prediction of survival in AD patients. E) Correlation between the proportion of myofibroblasts and sample age in unpublished data; Pearson’s r indicated and red line denotes linear regression with 95% confidence interval. F) Heatmap of Z-score of AA- and AD-associated genes across fibroblast subtypes. G) Gene Ontology enrichment analysis of pathways upregulated and downregulated in Fibro_C1_FBN1+ and myofibroblast subtypes. H) Regulatory network of transcription factors targeting *MYH11* and *ACTA2*; edge width and color denote regulatory importance, node size reflects degree centrality, arrows indicate regulatory direction. I) Regulatory network for transcription factors targeting *MYLK*, with same styling conventions for edge width, color, and node size. J) Matrix plot of inferred fibroblast subtype drug targets, identified via drug2cell. For panels a, c and i, meta-DEG analysis using random-effects model; log2FC indicates direction and magnitude of expression change; meta-P-value reflects pooled significance across studies.


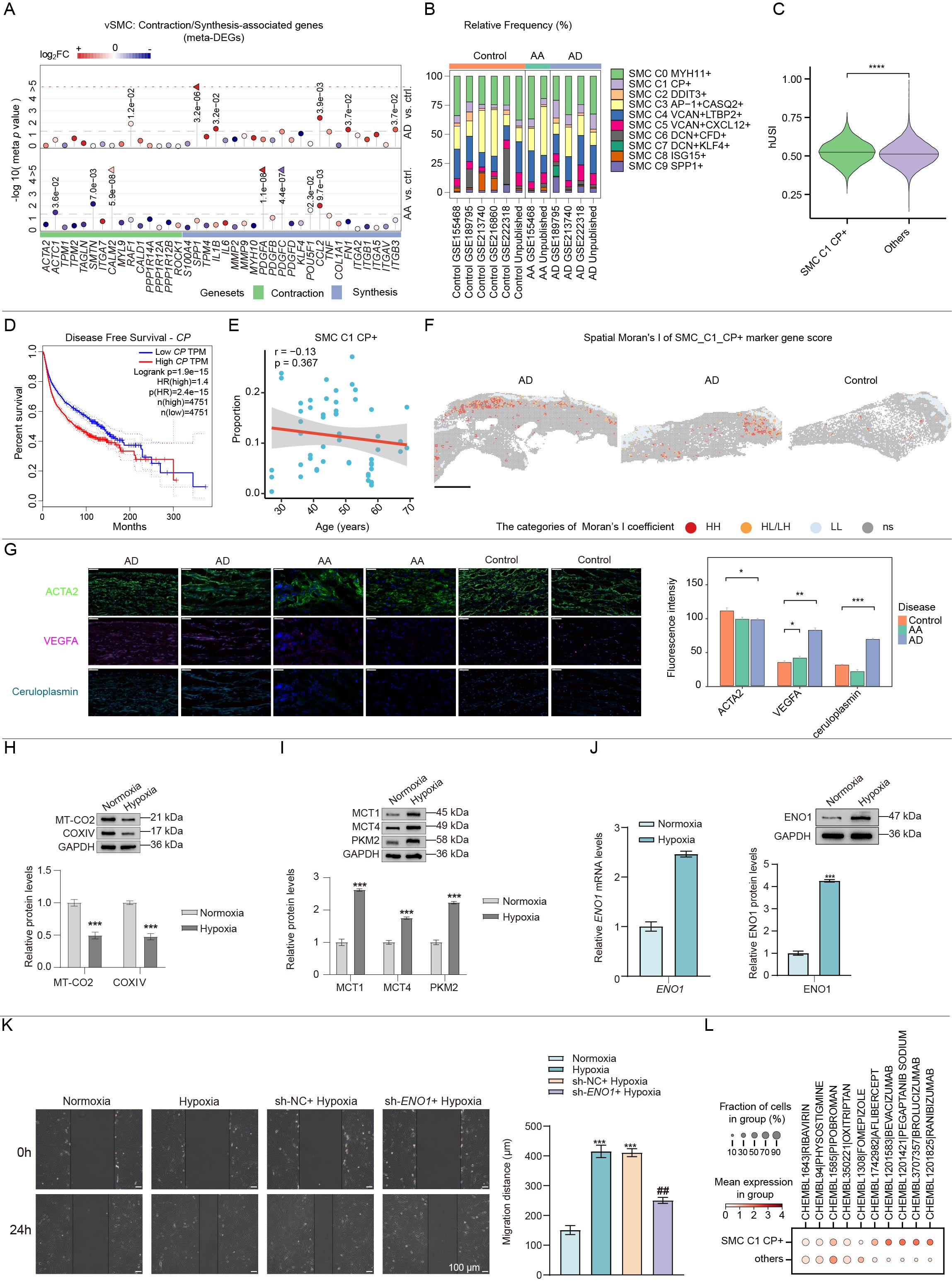


**Figure S6.** Functional and spatial profiling of vSMC subpopulations. A) Meta-analysis of expression differences in contractile and synthetic gene programs in vSMCs. B) Stacked bar plots showing frequency distributions of vSMC subtypes across disease states. C) hUSI scores across vSMC subtypes in the unpublished dataset, compared with the “Others” group using Wilcoxon rank-sum tests. Significance levels are indicated as *P < 0.05, **P < 0.01, ***P < 0.001, and ****P < 0.0001. D) Disease-free survival analysis of *CP* expression based on the TCGA pan-cancer cohort using the GEPIA platform (http://gepia.cancer-pku.cn/detail.php). All 32 cancer types available in the platform were included. This analysis is presented only as a general reference for prognostic gene-expression patterns and should not be interpreted as a direct prediction of survival in AD patients. E) Correlation between proportions of the SMC_C1_CP+ subtype and sample age in unpublished data; Pearson’s r is indicated, and the red line shows linear regression with a 95% confidence interval. F) Local Moran’s I hotspot localization for the SMC_C1_CP+ markers (*CP*, *NDRG1*, *VEGFA*) across human aortic tissues, identifying spatial clusters. Scale bars = 2 mm. G) Immunofluorescence images showing SMC_C1_CP+ markers (*ceruloplasmin*, *VEGFA*) in aortic tissues from AA, AD, and control samples; scale bars = 50 µm. Statistical comparisons via Wilcoxon rank-sum test; significance indicated as *P < 0.05, **P < 0.01, ***P < 0.001, ****P < 0.0001. H-J) Western blot and qPCR analyses of mitochondrial and metabolic markers: H), *MT-CO2*, *COXIV* under normoxia vs hypoxia; I), *MCT1*, *MCT4*, *PKM2* under normoxia vs hypoxia; J), *ENO1* expression levels assessed by qPCR and Western blot under both conditions. K), vSMC migration assessed by scratch wound-healing assays under experimental conditions. L), Matrix plot showing vSMC subtype–specific drug targets inferred via drug2cell.


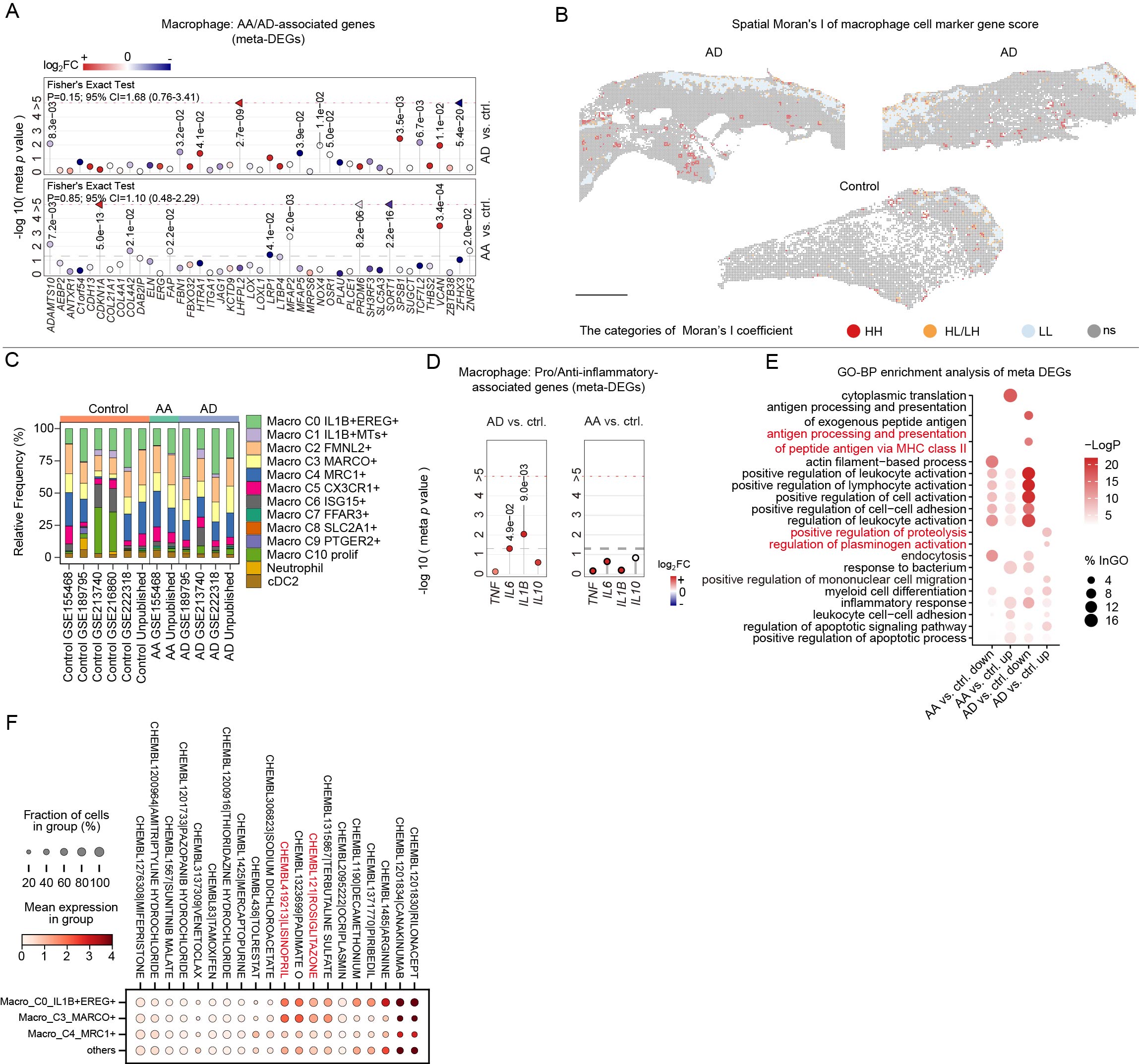


**Figure S7.** Characterization of macrophage subpopulations across disease conditions. A) Meta-analysis of AA- and AD-related gene expression differences in macrophages. B) Spatial hotspot localization in human aortic tissues across disease states using Local Moran’s I for macrophage marker gene score (*C1QA*, *C1QB*, *CD163*, *CD14*). Areas of significant local spatial autocorrelation (hotspots) are highlighted. Scale bars = 2 mm. C) Stacked bar plots showing the frequency distributions of macrophage subtypes in different disease states. D) Meta-analysis of differential expression of pro-inflammatory genes (*TNF*, *IL6*, *IL1B*) versus anti-inflammatory gene (*IL10*) in macrophages. E) Gene Ontology enrichment analysis of pathways upregulated or downregulated among macrophage-associated meta-DEGs across disease groups. F) Matrix plot of inferred drug targets across macrophage subtypes, identified by drug2cell. For panels B and G, DEGs were meta-analyzed via random-effects models; log2FC indicates both magnitude and direction of expression change, and meta-analysis P values reflect pooled significance across datasets.


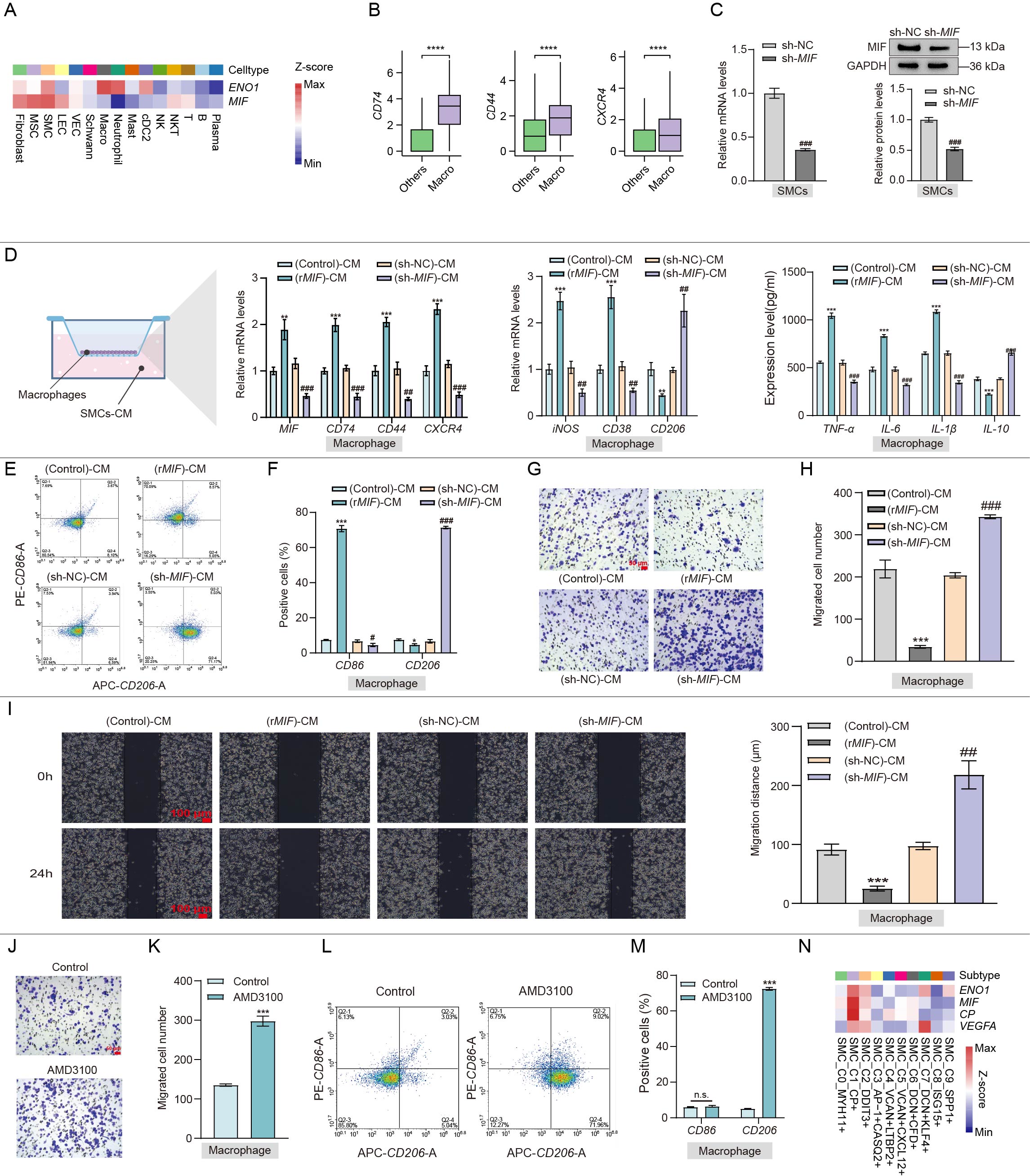


**Figure S8.** SMC-derived *MIF* promotes macrophage inflammatory polarization and retention in the aorta. A) Heatmap of *ENO1* and *MIF* Z-score across different cell types. B) Boxplots illustrating expression of *MIF* receptors (*CD74*, *CD44*, *CXCR4*) in macrophage versus non-macrophage cells. Pairwise comparisons by Wilcoxon rank-sum test; significance annotated as *P < 0.05, **P < 0.01, ***P < 0.001, ****P < 0.0001. C) *MIF* mRNA and protein expression in smooth muscle cells under sh-*MIF* knockdown, assessed by qPCR and Western blot. D) Expression levels of *MIF*, its receptors, and inflammatory markers in conditioned media and macrophage cultures under four conditions: Control (untreated), recombinant *MIF* (r*MIF*), sh-NC (control shRNA), and sh-*MIF* (*MIF* knockdown). E,F) Flow cytometry quantification of M2 (*CD206+CD86-*) versus M1 (*CD86+CD206-*) macrophage ratios following co-culture with conditioned media from hypoxic smooth muscle cells under various treatments. G,H) Transwell assays illustrating macrophage migration after exposure to conditioned media from hypoxic smooth muscle cells with different treatments. I) Scratch-wound healing assay assessing macrophage migration ability under Control, r*MIF*, sh-NC, and sh-*MIF* conditions following co-culture with conditioned media from hypoxic smooth muscle cells. J,K) Transwell assay of macrophage migration in the presence of the *CXCR4* antagonist AMD3100. L,M) Flow cytometry analyses of M2/M1 macrophage ratios following AMD3100 treatment. N) Heatmap of *ENO1* and *MIF* Z-score across different vSMC subtypes.
